# Circular RNA CRIM1 functions as a ceRNA to promote nasopharyngeal carcinoma metastasis and docetaxel chemoresistance through upregulating FOXQ1

**DOI:** 10.1186/s12943-020-01149-x

**Published:** 2020-02-15

**Authors:** Xiaohong Hong, Na Liu, Yelin Liang, Qingmei He, Xiaojing Yang, Yuan Lei, Panpan Zhang, Yin Zhao, Shiwei He, Yaqin Wang, Junyan Li, Qian Li, Jun Ma, Yingqin Li

**Affiliations:** State Key Laboratory of Oncology in South China, Collaborative Innovation Center of Cancer Medicine, Guangdong Key Laboratory of Nasopharyngeal Carcinoma Diagnosis and Therapy, Guangzhou, Sun Yat-sen University Cancer Center, Guangdong, 510060 China

**Keywords:** circCRIM1, ceRNA, Nasopharyngeal carcinoma, Metastasis, Chemoresistance

## Abstract

**Background:**

Circular RNAs (circRNAs), a new type of noncoding RNA (ncRNA), have been identified as significant gene expression regulators and are involved in cancer progression. However, the roles of circRNAs in nasopharyngeal carcinoma (NPC) remain largely unknown.

**Methods:**

Here, the expression profile of circRNAs in a pair of NPC cell lines with different metastatic abilities (S18 and S26 cells) was analyzed by RNA-sequencing. Quantitative reverse transcription PCR was used to detect the expression level of circCRIM1 in NPC cells and tissues. Then, function experiments in vitro and in vivo were performed to evaluate the effects of circCRIM1 on NPC metastasis and EMT. Mechanistically, RNA immunoprecipitation, luciferase reporter assay, pull-down assay with biotinylated miRNA, fluorescent in situ hybridization were performed to confirm the interaction between circCRIM1 and miR-422a in NPC. The clinical value of circCRIM1 was evaluated in NPC metastasis and chemosensitivity.

**Results:**

We identified that circCRIM1 was upregulated in highly metastatic NPC cells. CircCRIM1 was also overexpressed in NPC tissues with distant metastasis, and its overexpression promoted NPC cell metastasis and EMT. Mechanistically, circCRIM1 competitively bound to miR-422a and prevented the suppressive effects of miR-422a on its target gene FOXQ1, which finally led to NPC metastasis, EMT and docetaxel chemoresistance. Furthermore, high circCRIM1 expression was associated with unfavorable survival in NPC patients. We established a prognostic model based on circCRIM1 expression and N stage that effectively predicted the risk of distant metastasis and treatment response to docetaxel-containing induction chemotherapy in NPC patients.

**Conclusions:**

Our findings reveal the critical role of circCRIM1 specifically in promoting NPC metastasis and chemoresistance via a ceRNA mechanism and provide an exploitable biomarker and therapeutic target for prognosis and treatment resistance in NPC patients.

## Introduction

Nasopharyngeal carcinoma (NPC), which arises from the nasopharynx epithelium, is particularly prevalent in Southeast Asia, North Africa, the Middle East and Alaska [1, 2]. More than 70% of NPC patients are first diagnosed with locoregionally advanced disease because of the concealed symptoms and aggressiveness [3, 4]. According to the NCCN guidelines, radiotherapy combined with chemotherapy is the primary treatment strategy for locoregionally advanced NPC (LA-NPC) and substantially reduces locally recurrent disease [5]. However, distant metastasis is still the main reason for treatment failure and accounts for most cancer-related death from NPC [6]. Therefore, it is critical to further address the molecular mechanisms underlying NPC metastasis to develop more effective treatment options.

Circular RNAs (circRNAs) have emerged as a new, large class of noncoding RNAs characterized by covalently closed loop structures without 5′ caps and 3′ poly(A) tails [7, 8]. Although circRNAs were initially considered byproducts of splicing, high-throughput sequencing analysis has identified many circRNAs widely expressed in a tissue-specific or cell type-specific manner [9–11]. Accumulating studies demonstrate that circRNAs play important roles in multiple biological functions, such as cell proliferation, migration, invasion, and pluripotency [12–15]. Recently, circRNAs have been identified to be dysregulated in different types of cancers [14–17]. CircRNAs can function as competitive endogenous RNAs (ceRNAs) or protein-coding RNAs or interact with RNA-binding proteins to regulate the expression of genes involved in tumorigenesis and progression [14, 15, 17]. However, the functions and mechanisms of circRNAs remain to be elucidated in NPC.

Increasing evidence suggests that systemic induction chemotherapy (IC) can effectively eliminate NPC micrometastasis and ultimately improve patient survival [18–20]. However, the responses to chemotherapy are heterogeneous, and some patients can develop resistance, leading to treatment failure [20]. Currently, the recommended tumor-node-metastasis (TNM) staging system is not effective enough to predict ideal candidates for chemotherapy among NPC patients, which highlights the urgent need to explore new biomarkers for determining treatment [1, 6]. Recent studies have revealed that circRNAs have the potential to be diagnostic and prognostic markers or therapeutic targets for cancer due to their tissue-specific expression and biological function [14–17, 21]. Nevertheless, few reports have examined the association and regulatory mechanisms of circRNAs in drug resistance.

Here, using RNA sequencing, we identified circCRIM1 as a specific circRNA upregulated in NPC patients with distant metastasis. CircCRIM1 promotes NPC metastasis and docetaxel chemoresistance by functioning as a miR-422a sponge to upregulate FOXQ1 expression. Moreover, circCRIM1 expression is an independent prognostic factor and, when combined with N stage, can distinguish NPC patients with different risk of distant metastasis and response to docetaxel-based IC. Therefore, this study reveals that circCRIM1 exerts oncogenic potential and might be a promising marker to predict metastasis and chemotherapy benefits in NPC.

## Results

### CircRNA CRIM1 is upregulated in NPC patients with distant metastasis

**Fig. 1:**
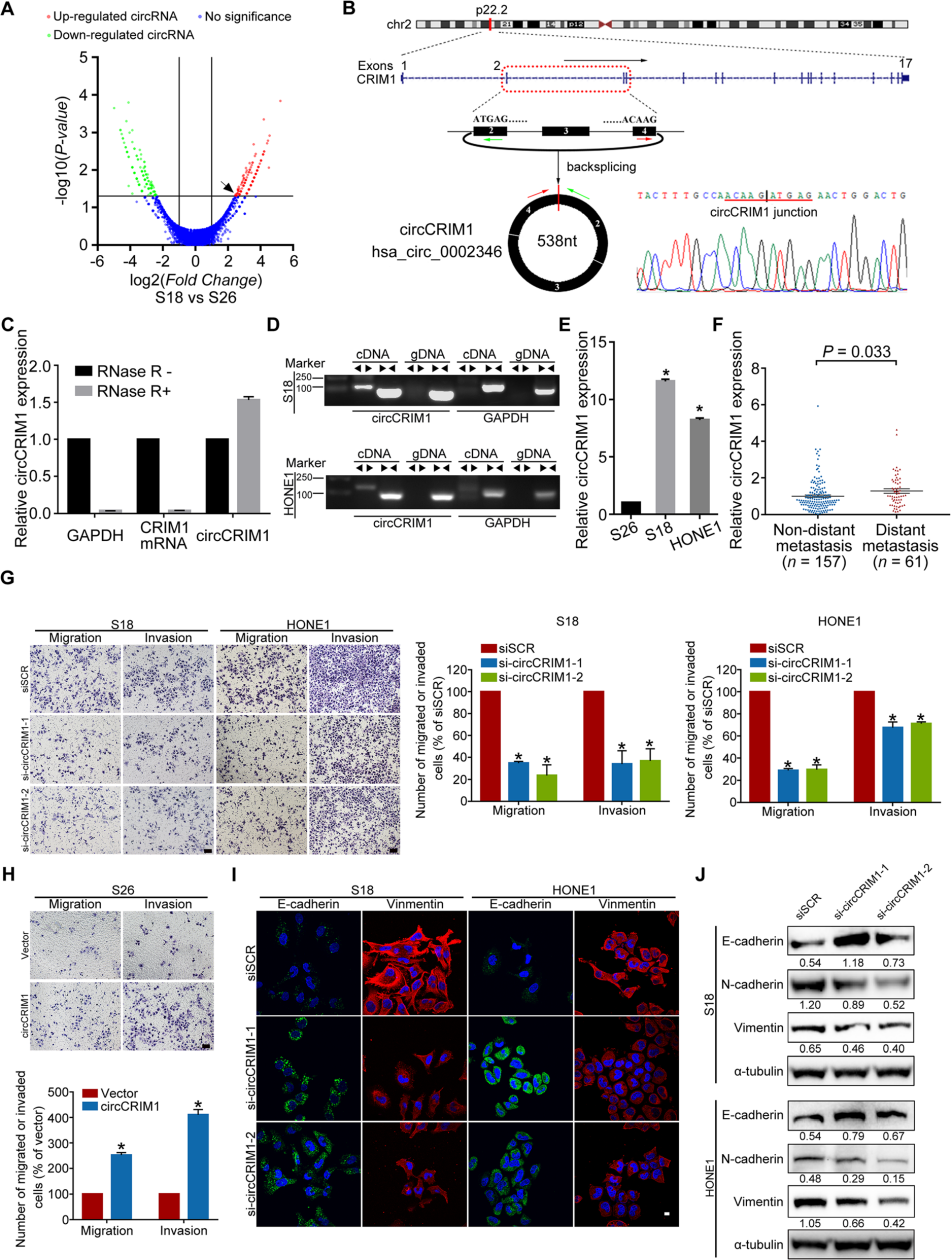
CircCRIM1 is upregulated in NPC patients with distant metastasis and promotes NPC cell metastasis and EMT in vitro. **a** Volcano plots of differential circRNAs between high metastasis S18 cells and low metastasis S26 cells. **b** Genomic loci of the circCRIM1 gene. The red and green arrows represent divergent primers. The backsplicing junction was validated by Sanger sequencing. **c** Quantitative real-time PCR (qRT-PCR) analysis of GAPDH, CRIM1 mRNA and circCRIM1 expression after RNase R treatment. **d** circCRIM1 expression in cDNA and genomic DNA (gDNA). GAPDH was used as a negative control. **e** Relative expression of circCRIM1 in S18, HONE1 and S26 cells. **f** Relative expression of circCRIM1 in NPC tissue without (*n* = 157) or with (*n* = 61) distant metastasis. **g-h** Representative and quantified results of the Transwell migration and invasion assays in NPC cells transfected with circCRIM1 siRNA or scrambled control (siSCR, **g**) or with circCRIM1 overexpression plasmid or vector (**h**). Scale bar, 100 μm. **i** Immunofluorescence images of E-cadherin and Vimentin expression in NPC cells with or without circCRIM1 downregulation. Scale bar, 10 μm. **j** Western blotting analysis of E-cadherin, N-cadherin and Vimentin in NPC cells with or without circCRIM1 depletion. The data are presented as the mean ± S.D. All in vitro data are representative of three independent experiments. Student’s *t*-test; *P < 0.05

To investigate metastasis-associated circRNAs in NPC, we performed an RNA sequencing analysis of ribosomal RNA-depleted and RNase-R-treated RNA from a pair of NPC cell lines with different metastatic abilities (S18 cells with high metastasis potential and S26 cells with low metastasis potential). Differentially expressed circRNAs were found between the S18 and S26 cell lines (Fig. 1a). In total, 56 circRNAs were upregulated, whereas 28 circRNAs were downregulated in the highly metastatic S18 cell line (|fold changes| ≥ 2 and FDR < 0.05). Hsa_circ_0002346 (chr2: 36623756–36,669,878) was one of the most upregulated circRNAs. By mapping the human reference genome (GRCh37/hg19), we identified that hsa_circ_0002346 is derived from exons 2, 3 and 4 of the *CRIM1* gene, which is located on chromosome 2p22.2. Based on known circRNA database (circBase) [22], 107 circRNAs are derived from the *CRIM1* gene and 15 isoforms could be detected in S18 NPC cell line (Additional file 1: Figure S1a-c, Table S1 and Additional file 2). Since hsa_circ_0002346 was the first reported isoform of circRNAs arise from the *CRIM1* gene, we termed it as “circCRIM1”, as previously described (Fig. 1b) [23–25].

We designed divergent junction-specific primers that spanned the backsplicing junction of circCRIM1 to detect its expression by quantitative real-time PCR (qRT-PCR). Sanger sequencing of the amplified PCR products confirmed the backsplicing sites of circCRIM1 and distinguished circCRIM1 from a lariat RNA (Fig. 1b) [26]. To check its resistance to RNase R digestion, total RNA was treated with RNase R and the linear isoform levels were used to illustrate the efficacy of RNase R treatment. The results showed that circCRIM1 was resistant to RNase R, further proving the circular structure of circCRIM1 in NPC cells (Fig. 1c). In addition, convergent primers and divergent primers were designed to amplify CRIM1 mRNA and circCRIM1 using cDNA and genomic DNA (gDNA) from NPC cells. The results showed that circCRIM1 could be detected in only cDNA and not in gDNA (Fig. 1d).

To verify the RNA sequencing results, we performed qRT-PCR to examine circCRIM1 expression in NPC cell lines and clinical tissues. Consistently, circCRIM1 expression was significantly higher in S18 and another NPC cell line, HONE1, than in S26 cell line (Fig. 1e, *P* < 0.05). Although circCRIM1 levels were relatively lower than the housekeeping mRNA GAPDH, it was as abundant as the CRIM1 linear mRNA, especially in S18 cells (Additional file 1: Figure S1d). Compared with normal nasopharynx tissues (*n* = 16), circCRIM1 was significantly upregulated in NPC tissues (*n* = 20, Additional file 1: Figure S1e). Moreover, circCRIM1 expression was particularly increased in NPC patients with distant metastasis (*n* = 218, Fig. 1f, *P* < 0.05). Taken together, these findings demonstrate that circCRIM1 is specifically upregulated in NPC patients with distant metastasis.

### CircCRIM1 promotes NPC cell migration, invasion and EMT in vitro

To evaluate the potential role of circCRIM1 in NPC metastasis, we designed two short interfering RNAs (siRNAs) to specifically target the backsplicing site of circCRIM1 but not change linear CRIM1 mRNA expression (Additional file 1: Figure S2a-b). We also generated a circCRIM1 overexpression vector to transfect the low metastasis NPC cell line S26. CircCRIM1 was successfully overexpressed in S26 cells, while CRIM1 mRNA showed no obvious change (Additional file 1: Figure S2c). Transwell assays revealed that knocking down circCRIM1 expression significantly suppressed the migration and invasion abilities of S18 and HONE1 cells (Fig. 1g, *P* < 0.05). To prove that these were on-target effects resulting from circCRIM1 knockdown, we performed sequence alignment analyses and constructed a circCRIM1 overexpression vector resistant to siRNAs with mutated back-spliced junction (circCRIM1-si-mut), according to previous instructions [24, 25, 27]. Although there were 15 circRNA isoforms derived from *CRIM1* gene in S18 cell line, only the isoform concerned in our study (circCRIM1/hsa_circ_0002346) contained completely complementary backsplice sequences to si-circCRIM1–1 and si-circCRIM1–2 (Additional file 1: Figure S3–S4). Moreover, the rescued experiments showed that ectopic expression of circCRIM1-si-mut fully rescued the suppressive effects after knockdown of endogenous circCRIM1, and promoted NPC cell migration and invasion (Additional file 1: Figure S5, *P <* 0.05). Meanwhile, ectopic circCRIM1 expression markedly promoted S26 cell migration and invasion (Fig. 1h, *P* < 0.05). Thus, these data suggest that circCRIM1 promotes NPC cell migratory and invasive abilities in vitro.

Notably, when circCRIM1 expression was knocked down in S18 and HONE1 cells, the NPC cell morphology changed from a spindle-shaped or elongated mesenchymal form to an epithelial form (Additional file 1: Figure S6a-b), which indicated that circCRIM1 may function to induce NPC cell EMT. It is known that EMT allows cells to acquire migratory and invasive behaviors during cancer metastasis. Next, we performed immunofluorescent staining and Western blotting assays to examine the effect of circCRIM1 on epithelial and mesenchymal marker expression. The results showed that circCRIM1 downregulation remarkably increased epithelial marker (E-cadherin) expression but decreased mesenchymal marker (N-cadherin and Vimentin) expression in NPC cells (Fig. 1i-j and Additional file 1: Figure S6c-d). Together, these results suggest that circCRIM1 induces NPC cell migration, invasion and EMT.

### CircCRIM1 serves as a sponge for miR-422a in NPC

**Fig. 2:**
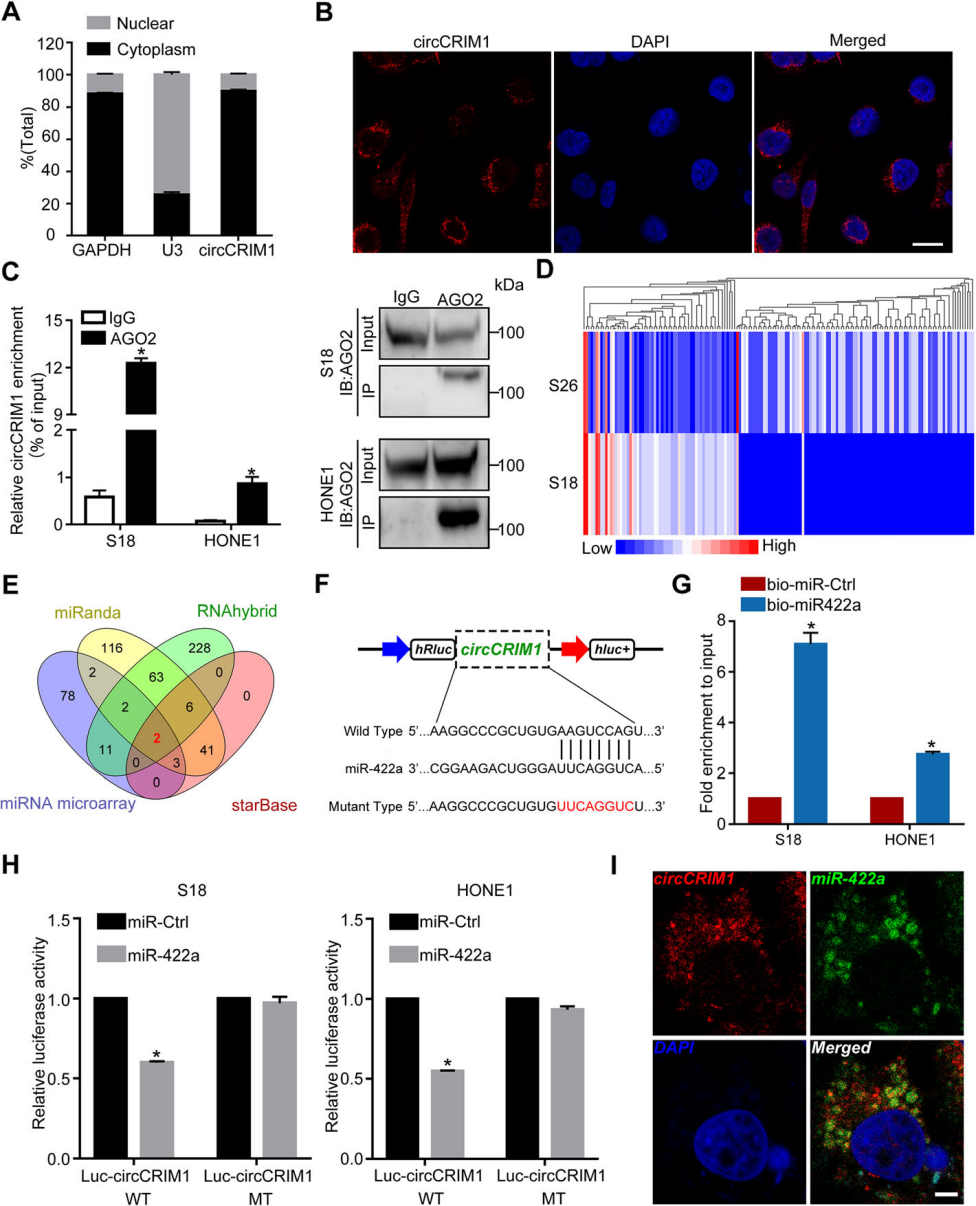
CircCRIM1 binds directly to miR-422a in NPC. **a** Levels of circCRIM1 in the nuclear and cytoplasmic fractions of S18 cells. **b** RNA fluorescence in situ hybridization (FISH) for circCRIM1. The nuclei were stained with DAPI. Scale bar, 20 μm. **c** RNA immunoprecipitation (RIP) analysis of circCRIM1 in S18 and HONE1 cells using antibodies against AGO2. Western blotting analysis of immunoprecipitated AGO2 protein is shown. **d** Clustered heatmap of the differentially expressed miRNAs in S18 and S26 cells. **e** Overlapping of the target miRNAs of circCRIM1 based on miRNA microarray, miRanda and RNAhybrid algorithms, and StarBase. **f** Schematic of the predicted miR-422a binding site on circCRIM1. **g** Enrichment of circCRIM1 in S18 and HONE1 cells after pull-down assay with biotinylated miR-422a. Mean (*n* = 3) ± S.D. Student’s *t*-tests; *P < 0.05. **h** Luciferase activity of wild type or mutated circCRIM1 in NPC cells after cotransfection with miR-422a or miRNA control. **i** Colocalization between miR-422a and circCRIM1 was observed by RNA FISH in S18 cells. The nuclei were stained with DAPI. Scale bar, 10 μm. The data are presented as the mean (*n* = 3) ± S.D. Student’s *t*-test; *P < 0.05

To observe the cellular distribution of circCRIM1, we performed qRT-PCR and FISH assays for nuclear and cytoplasmic circCRIM1 RNA. The results showed that circCRIM1 transcripts were located predominantly in the cytoplasm (Fig. 2a-b). Previous studies have reported that circRNAs can function as miRNA sponges to regulate gene expression [8]. Given that circCRIM1 was enriched in the cytoplasm, we further explored whether circCRIM1 served as a platform for silencing complex (RISC) catalytic subunit Argonaute 2 (AGO2) and acted as a ceRNA in the pathogenic process of NPC. RNA immunoprecipitation (RIP) analysis revealed that circCRIM1 was significantly enriched by the AGO2 antibody (Fig. 2c, *P* < 0.05), suggesting that circCRIM1 may possess miRNA-related functions.

To determine potential miRNAs that interact with circCRIM1, we next conducted a microarray analysis to evaluate the miRNA expression profiles of S18 and S26 NPC cells. The results revealed 63 upregulated and 98 downregulated miRNAs in the high metastasis S18 cell line (|fold changes| ≥3 and FDR < 0.05, Fig. 2d and Additional file 1: Table S2). Subjecting downregulated miRNAs to the bioinformatic algorithms miRanda, RNAhybrid and StarBase [28–30] to predict miRNAs revealed that miR-422a and miR-4436a contain putative targeting sites for the circCRIM1 region (Fig. 2e-f and Additional file 1: Table S3–S4). We then performed luciferase assays to validate the binding capability of these two miRNAs to circCRIM1. A miR-422a or miR-4436a mimic was cotransfected with circCRIM1 luciferase reporters into NPC cells. The results showed that the luciferase activity was significantly reduced by approximately 40% after miR-422a overexpression but not after miR-4436a overexpression (Additional file 1: Figure S7a). We then mutated the target sites for miR-422a in the circCRIM1 luciferase reporter (Fig. 2f). Ectopic miR-422a expression significantly reduced the luciferase activity of the circCRIM1 wild type reporter but not the mutated reporter in NPC cells (Fig. 2h, *P* < 0.05). Furthermore, pull-down assays revealed significantly higher circCRIM1 enrichment by biotin-miR-422a (Fig. 2g, *P* < 0.05). Moreover, the double FISH assay indicated circCRIM1 and miR-422a colocalization in NPC cells (Fig. 2i). All these findings prove that circCRIM1 can bind directly to miR-422a.

### FOXQ1 is a downstream target of miR-422a and circCRIM1 and is involved in metastasis and docetaxel chemoresistance

**Fig. 3:**
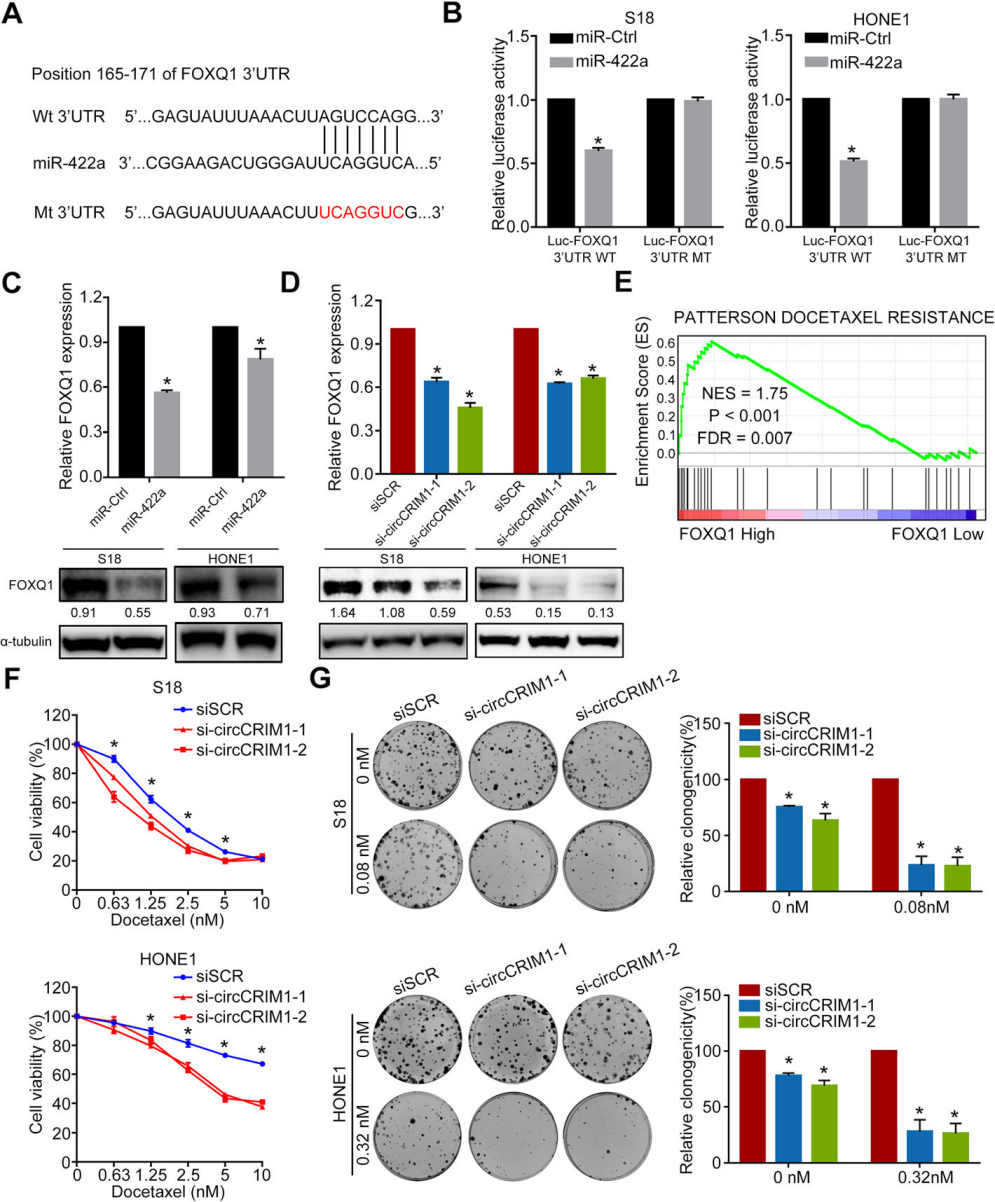
FOXQ1 is a direct target of miR-422a and is involved in circCRIM1-associated NPC metastasis and chemoresistance. **a** Schematic of the predicted miR-422a binding site in the FOXQ1 3′ UTR. **b** Luciferase activity of wild type or mutated FOXQ1 3′ UTR in NPC cells after cotransfection with miR-422a or control. **c-d** qRT-PCR (**c**) and Western blotting (**d**) of FOXQ1 expression in NPC cells with or without miR-422a overexpression. **e** Docetaxel resistance-related biological functions were enriched in response to high FOXQ1 expression (GSE12452). **f-g** Dose-response curves (**f**) and representative and quantified results (**g**) for docetaxel treatment in S18 and HONE1 cells with or without circCRIM1 downregulation. The cells were treated with low-dose docetaxel. The error bars represent the mean (*n* = 3) ± S.D. Student’s *t*-test; *P < 0.05

Furthermore, we employed the miRDB [31] algorithm to explore the common downstream targets of circCRIM1 and miR-422a. Forkhead box Q1 (FOXQ1) was predicted as a putative target gene of miR-422a. To confirm these findings, we constructed a luciferase reporter vector with the wild type or mutated FOXQ1 3′ UTR binding site for miR-422a (Fig. 3a). The luciferase activities of the FOXQ1 3′ UTR wild type reporter were significantly reduced in NPC cells transfected with miR-422a mimic (Fig. 3b, *P* < 0.05). However, no significant difference in luciferase activity was noted for scrambled control and miR-422a mimic when cotransfected with the mutated FOXQ1 3′ UTR reporter (Fig. 3b). qRT-PCR and Western blotting analysis revealed that miR-422a overexpression significantly reduced FOXQ1 mRNA and protein levels (Fig. 3c and Additional file 1: Figure S2d). Similarly, circCRIM1 depletion remarkably repressed FOXQ1 expression (Fig. 3c-d). Conversely, circCRIM1 overexpression significantly increased FOXQ1 expression (Additional file 1: Figure S7b). These data indicate that FOXQ1 can be regulated by miR-422a and circCRIM1.

To evaluate the effects of FOXQ1 on NPC phenotypes, we performed GSEA to compare gene profiles of NPC samples with high and low FOXQ1 expression using published NPC datasets (GSE12452). The results showed that compared with FOXQ1-low NPCs, FOXQ1-high NPCs were significantly enriched in gene sets related to metastasis (NAKAMURA_METASTASIS), which is consistent with the promoting role of circCRIM1 in NPC metastasis (Additional file 1: Figure S7c). Moreover, chemoresistance (Patterson_Docetaxel_Resistance) was enriched in NPC patients with high FOXQ1 expression (Fig. 3e). We also investigated whether circCRIM1 regulates chemosensitivity to docetaxel, which was previously shown to be an effective IC agent that controls metastasis in our randomized controlled phase 3 clinical trial. CCK8 and colony formation assays revealed that circCRIM1 depletion significantly enhanced the sensitivity of NPC cells to docetaxel (Fig. 3f-g, *P* < 0.05). The IC50 values of docetaxel significantly decreased to 1.451 and 1.018 nM/L in S18 cells, 5.034 and 5.076 nM/L in HONE1 cells with circCRIM1 downregulation, compared with those in control cells (2.176 nM/L in S18 cells and 24.78 nM/L in HONE1 cells, Fig. 3f). Collectively, FOXQ1 may exert functions similar to circCRIM1 to promote NPC cell metastasis and docetaxel chemoresistance.

### CircCRIM1 promotes NPC cell metastasis and EMT by relieving the suppression effects of miR-422a on FOXQ1

**Fig. 4:**
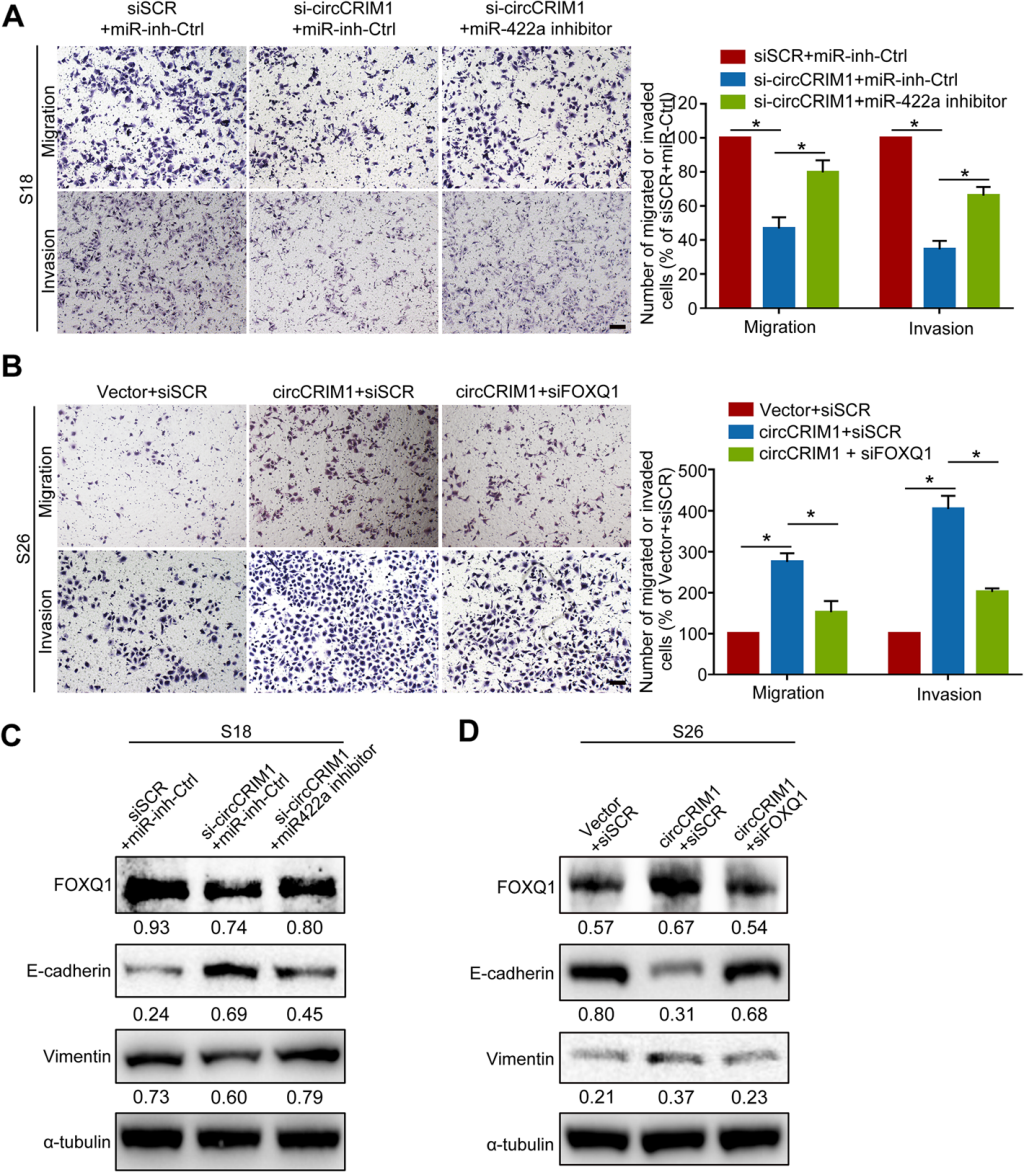
CircCRIM1 promotes NPC cell metastasis and EMT by relieving miR-422a repression of FOXQ1 expression. **a** Representative and quantified results of the Transwell migration and invasion assays for S18 cells cotransfected with either si-circCRIM1 or scrambled control and miR-422a inhibitor or miRNA inhibitor control. **b** Representative and quantified results of the Transwell assays for S26 cells cotransfected with either circCRIM1 overexpression plasmid or vector and FOXQ1 siRNA or scrambled control. **c-d** Western blotting analysis of FOXQ1, E-cadherin, Vimentin and α-tubulin expression. Mean (*n* = 3) ± S.D. Student’s *t*-tests; *P < 0.05. Scale bar, 100 μm

To confirm whether circCRIM1 exerts its promoting effect through miR-422a, Transwell assays were performed after miR-422a inhibitor transfection to knockdown its expression. We observed that miR-422a knockdown could significantly restore the suppressive effects of circCRIM1 downregulation on NPC cell migratory and invasive abilities (Fig. 4a). In addition, miR-422a inhibition markedly abrogated the inhibition of FOXQ1 expression and EMT induced by circCRIM1 downregulation, as evidenced by impaired epithelial marker expression (E-cadherin) and increased mesenchymal marker expression (Vimentin, Fig. 4c). Therefore, these findings suggest that circCRIM1 regulates FOXQ1 expression and facilitates NPC cell migration and invasion through miR-422a.

We next investigated whether FOXQ1 is required for the promoting effects of the circCRIM1-miR-422a axis on NPC cells. The results showed that FOXQ1 depletion substantially rescued the enhanced NPC cell migration and invasion caused by circCRIM1 overexpression (Fig. 4b). Moreover, FOXQ1 inhibition remarkably reversed the increased FOXQ1 expression and EMT induced by exogenous circCRIM1 manipulation in NPC cells (Fig. 4d). Taken together, these results reveal that circCRIM1 serves as a sponge for miR-422a to regulate FOXQ1 expression and promotes NPC cell metastasis and EMT via a ceRNA mechanism.

### CircCRIM1 promotes NPC cell metastasis and docetaxel chemoresistance in vivo

**Fig. 5:**
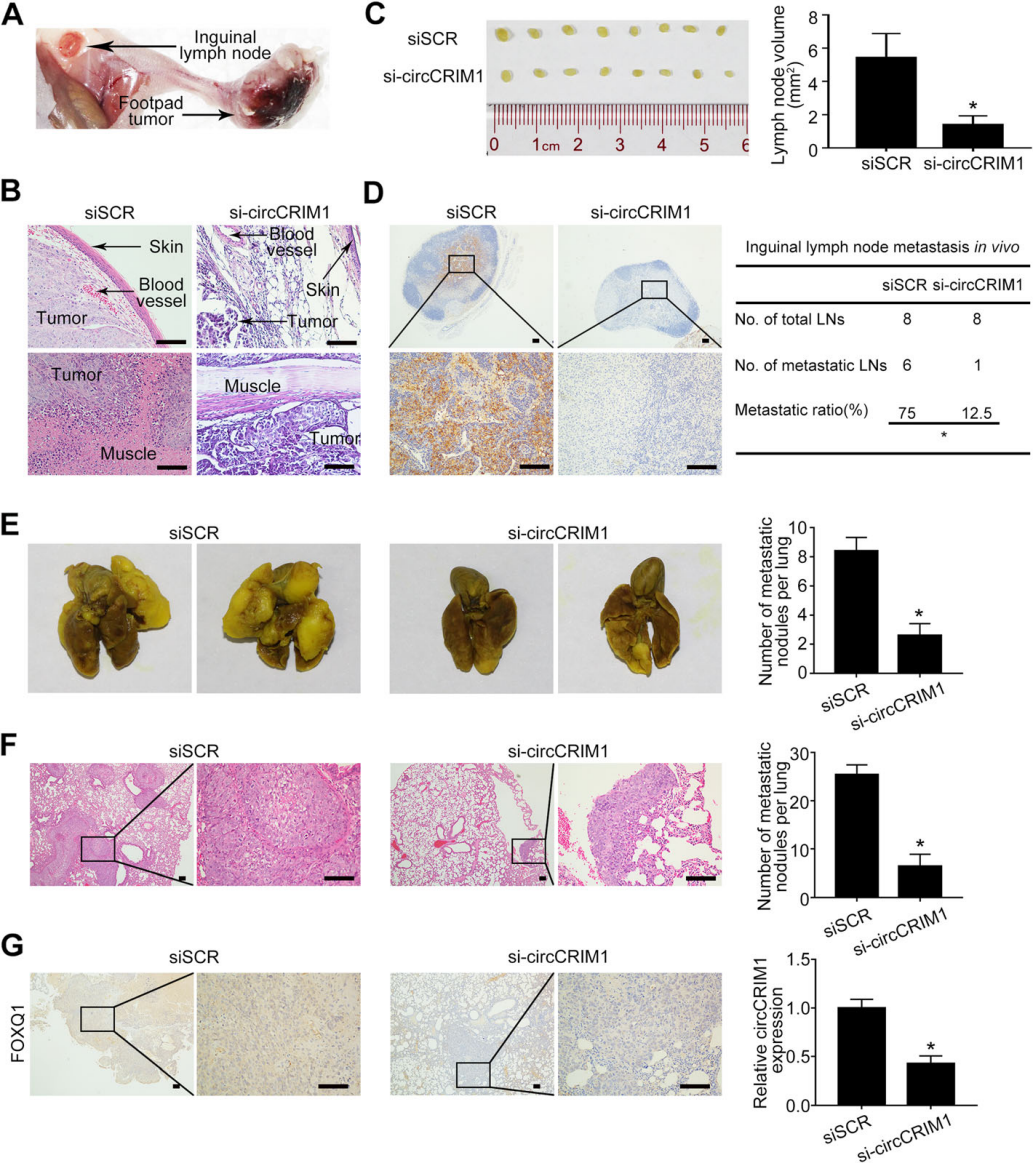
CircCRIM1 promotes NPC cell metastasis in vivo. **a** S18 cells were inoculated into mouse footpads. Representative images of footpad tumors after cholesterol-conjugated circCRIM1 siRNA or siSCR injection. **b** Representative microscopy images of footpad tumors. Scale bar, 100 μm. **c** Representative image and quantification of the volumes of the inguinal lymph nodes. Mean (*n* = 8) ± S.D. Student’s *t*-tests; *P < 0.05. **d** Representative images of pan-cytokeratin positive inguinal lymph nodes and metastatic ratios. Scale bar, 100 μm. Inguinal lymph node metastatic ratios. Chi-square test.; *P < 0.05. **e-f** Representative images and quantification of macroscopic (**e**) and microscopic metastatic nodules (**f**) in the lungs of mice after systemic administration of cholesterol-conjugated circCRIM1 siRNA or NC control via tail vein injection. Mean (*n* = 5) ± S.D. Student’s *t*-test, *P < 0.05. Scale bar, 100 μm. **g** Representative images of immunohistochemical staining for FOXQ1 expression and relative circCRIM1 expressions in the lungs of mice. Scale bar, 100 μm. Mean (*n* = 5) ± S.D. Student’s *t*-test, *P < 0.05

To evaluate the effect of circCRIM1 on NPC metastasis in vivo, we constructed an inguinal lymph node metastasis model by transplanting S18 cells into the foot pads of nude mice (*n* = 8 per group). After two weeks, cholesterol-conjugated circCRIM1 siRNA (si-circCRIM1–1) was injected locally into the mouse footpad tumor twice a week for two weeks. The primary tumors and inguinal lymph nodes were dissected and analyzed (Fig. 5a). Hematoxylin and eosin (H&E) staining showed that the footpad tumors in the circCRIM1 depletion group exhibited less aggressive phenotypes with invasion towards the skin and muscle (Fig. 5b). Strikingly, circCRIM1 downregulation significantly reduced the volumes and ratios of metastatic inguinal lymph nodes and the number of pan-cytokeratin-positive tumor cells (Fig. 5c-d, *P* < 0.05).

We next generated a lung metastatic colonization model by inoculating S18 cells into the tail veins of mice (*n* = 5 per group). Two weeks after model construction, cholesterol-conjugated si-circCRIM1 was administered to the mice by tail vein injection twice a week for five weeks. As shown in Fig. 5e, systemic administration of si-circCRIM1 resulted in a remarkable reduction of lung metastatic nodules in vivo (Fig. 5e, *P* < 0.05). H&E staining confirmed that mice with circCRIM1 depletion exhibited significantly fewer and smaller metastatic tumor nodules in the lungs (Fig. 5f, *P* < 0.05). Of note, concordantly low levels of circCRIM1 and FOXQ1 expression were found in lung metastatic nodules in the circCRIM1-knockdown group in vivo (Fig. 5g). Consistent with the suppressive effects of circCRIM1 knockdown, FOXQ1 downregulation significantly reduced lung metastatic tumors in mice (Additional file 1: Figure S8, *P* < 0.05). Furthermore, we evaluated the effects of circCRIM1 on docetaxel treatment. The results showed that circCRIM1 downregulation significantly enhanced the docetaxel efficacy to treat NPC cell metastasis (Additional file 1: Figure S9, *P* < 0.05). Altogether, these findings suggest that circCRIM1 promotes NPC cell metastasis and docetaxel chemoresistance by regulating FOXQ1 in vivo.

### CircCRIM1 is associated with an adverse prognosis for distant metastasis in patients with NPC

**Fig. 6:**
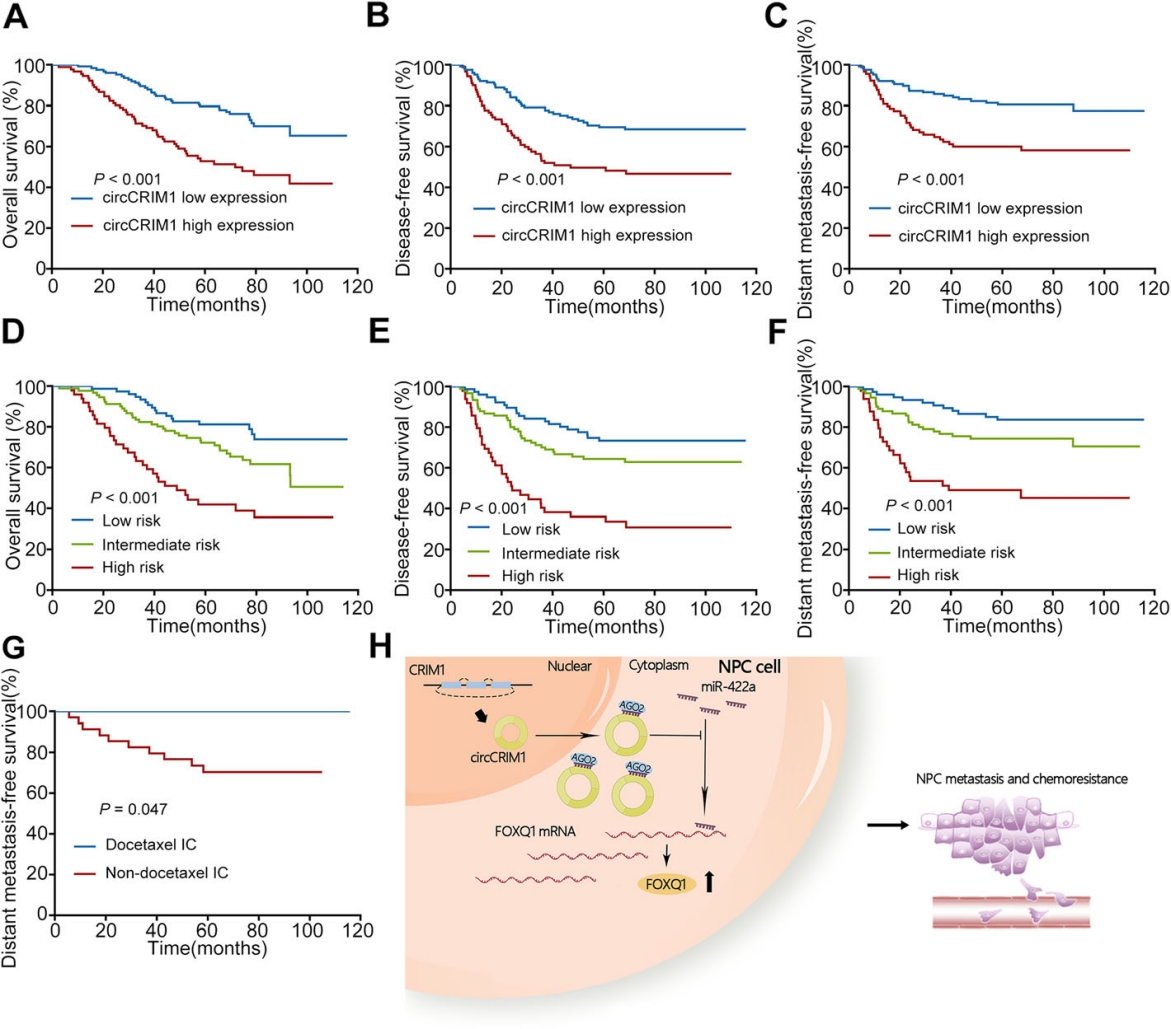
CircCRIM1 is associated with unfavorable prognosis and docetaxel-based IC resistance in patients with NPC. **a-c** Kaplan-Meier analysis of overall survival (OS, **a**), disease-free survival (DFS, **b**) and distant metastasis-free survival (DMFS, **c**) in NPC patients with low and high circCRIM1 expression. **d-f** OS (**d**), DFS (**e**) and DMFS (**f**) in NPC patients with low risk (low circCRIM1 expression and early N stage), intermediate risk (high circCRIM1 expression or advanced N stage) and high risk (high circCRIM1 expression and advanced N stage) according to the prognostic prediction model. **g** DMFS in low risk patients receiving IC with or without docetaxel. *P* values were determined using the log-rank test. **h** Hypothetical model for circCRIM1 function in NPC. CircCRIM1 acts as a sponge of miR-422a to promote NPC metastasis and docetaxel chemoresistance by relieving FOXQ1 expression

To evaluate the clinical value of circCRIM1 in NPC, we performed qRT-PCR to determine circCRIM1 expression in 218 NPC tissues. Based on the ROC analysis, 127 (58%) patients had low circCRIM1 expression, and 91 (42%) had high circCRIM1 expression. As shown in Additional file 1: Table S5, there were no significant associations between circCRIM1 expression and age, sex, WHO type, VCA-IgA, EA-IgA, T stage or TNM stage (all *P* > 0.05). However, circCRIM1 levels were significantly correlated with N stage (*P* = 0.034), death (*P* < 0.001) and distant metastasis (*P* = 0.001). Kaplan–Meier survival analysis showed that patients with high circCRIM1 expression had poorer overall survival (OS, *P* < 0.001), disease-free survival (DFS, *P* < 0.001) and distant metastasis-free survival (DMFS, *P* < 0.001) than those with low circCRIM1 expression (Fig. 6a-c).

Furthermore, multivariate Cox regression analysis found that circCRIM1 expression (OS, hazard ratio (HR), 2.134; 95% confidence interval (CI), 1.364–3.339; *P* = 0.001; DFS, HR, 1.915; 95% CI, 1.248–2.941; *P* = 0.003; DMFS, HR, 2.107; 95% CI, 1.258–3.528; *P* = 0.005) and N stage (OS, HR, 1.943; 95% CI, 1.243–3.039; *P* = 0.004; DFS, HR, 2.215; 95% CI, 1.433–3.423; *P* < 0.001; DMFS, HR, 2.272; 95% CI, 1.348–3.829; *P* = 0.002) were independent prognostic indicators for NPC (Additional file 1: Table S6). Taken together, circCRIM1 expression is clinically associated with unfavorable clinical outcomes for distant metastasis in NPC patients.

### Prognostic model combining circCRIM1 expression and N stage for predicting NPC metastasis and docetaxel IC benefit

To stratify patients with different risks of distant metastasis, we constructed a prognostic prediction model based on circCRIM1 expression status and N stage. Patients were classified into 3 groups: 77 (35.3%) patients in the low risk group (low circCRIM1 expression and early N stage), 92 (42.2%) patients in the intermediate risk group (high circCRIM1 expression or advanced N stage) and 49 (22.5%) patients in the high risk group (high circCRIM1 expression and advanced N stage). Patients in these 3 groups experienced significantly different risks of death, recurrence and distant metastasis (Fig. 6d-f).

Furthermore, we evaluated the prediction value of circCRIM1 expression for docetaxel-containing IC sensitivity using a prognostic model based on circCRIM1 expression status and N stage. In the low risk group, patients who received docetaxel IC exhibited significantly better distant metastasis control than patients who did not (5-year DMFS; 100% vs. 71.4%, *P* = 0.047, Fig. 6g). In contrast, patients with intermediate or high risk did not benefit from docetaxel-containing IC (Additional file 1: Figure S10). These findings indicate that the combination of circCRIM1 expression with N stage can be a promising signature to distinguish patients with different risk of metastasis and therapeutic responses to docetaxel-based IC.

Taken together, our study revealed that circCRIM1 exerts its function as a ceRNA through sponging miR-422a to regulate downstream FOXQ1 expression, and therefore contributes to metastasis and docetaxel-based IC chemoresistance in NPC (Fig. 6h).

## Discussion

In this study, we first identified circCRIM1 as a significantly upregulated circRNA in distant-metastatic NPC patients. Second, overexpression of circCRIM1 promoted NPC cell metastasis and docetaxel chemoresistance by competitively binding to miR-422a and then relieving the inhibitory effects on FOXQ1. Third, using a prognostic model with low circCRIM1 expression and early N stage, we stratified NPC patients at low risk of distant metastasis who could benefit from docetaxel-based IC. The current study reveals the vital roles and mechanisms of circCRIM1 and highlights the prognostic and therapeutic importance of circCRIM1 in NPC metastasis and chemoresistance.

As a novel class of noncoding RNA, circRNAs have been proven to play important roles in multiple biological processes, such as spermatogenesis, neural development, cell pluripotency disease initiation and progression [13, 32, 33]. It is evident that circRNAs are dysregulated and function as oncogenes or tumor suppressors in different cancer types with tissue-specific and cell-specific patterns [14, 15, 34, 35]. However, only a few circRNAs have been characterized, and the role of circRNA in NPC metastasis is largely unknown. Here, we identified that circCRIM1 was upregulated in specifically NPC cell lines with high metastasis potential and NPC patients with distant metastasis. Functional experiments confirmed that elevated circCRIM1 expression promoted NPC cell metastasis and contributed to docetaxel chemoresistance, revealing circCRIM1 as a potential oncogene in NPC. However, a previous study reported a conversely suppressive phenotype of circCRIM1 in lung adenocarcinoma metastasis [36]. These findings indicate that circCRIM1 could exert different functions in tumors with tissue or cell type-specific patterns, which is consistent with the specificity of circRNAs in different types of cancers [10, 37, 38]. We then focused on the underlying mechanism and clinical value of increased circCRIM1 expression in NPC.

CircRNAs were initially shown to function as miRNA sponges in a ceRNA mechanism and thereby competitively regulate miRNA targets [8, 15]. Recent studies have reported that some dysregulated circRNAs can absorb miRNA to modulate the expression of oncogenes or tumor suppressor genes in carcinogenesis and cancer development [15, 34, 35]. However, numerous circRNAs with low abundance are incompetent at sponging miRNAs [39]. It has been suggested that circRNAs with their stable and high expression in the cytoplasm are ideal ceRNAs [8]. In our study, circCRIM1 was highly expressed with over 0.1% GAPDH in NPC cells and predominantly located in the cytoplasm, which made a candidate miRNA sponge. Further experimental evidence revealed that circCRIM1 could directly bind to miR-422a and inhibit its activity. A previous study showed that miR-422a was downregulated and may serve as a tumor suppressor in several cancers, such as hepatocellular carcinoma, glioblastoma and gastric cancer [40–42]. However, the function of miR-422a in NPC is unknown. Our functional studies demonstrated that circCRIM1 knockdown could inhibit NPC cell migration and invasion, and this effect was attenuated by a miR-422a inhibitor. Our findings suggest, for the first time, that miR-422a is a functional target in the ceRNA network of circCRIM1 in NPC.

FOXQ1, a Forkhead box-containing transcription factor, is reported to play important roles in cell proliferation, motility, EMT and stemness [43–45]. Previous studies have demonstrated that FOXQ1 is increased and functions as an oncogene in several cancers, such as hepatocellular carcinoma, breast cancer and colorectal cancer [43–45]. However, there is little knowledge regarding the role and regulatory mechanism of FOXQ1 in NPC. It is known that miRNAs can regulate gene expression by directly binding to the 3′ UTR of target mRNAs and leading to mRNA degradation or translation inhibition. In our study, we revealed that FOXQ1 was a direct target of miR-422a. Furthermore, we demonstrated that circCRIM1 upregulated FOXQ1 expression by relieving the posttranscriptional suppression capabilities of miR-422a. Functionally, FOXQ1 depletion impaired the stimulating effects of circCRIM1 on NPC cell metastasis and EMT. Therefore, we uncovered a new mechanism by which circCRIM1 functions as a ceRNA and weakens the endogenous inhibitory effects of miR-422a on FOXQ1 in NPC.

Recently, the potential of circRNAs as cancer biomarkers and therapy targets has attracted much attention [21, 35]. We discovered that high circCRIM1 expression was associated with unfavorable clinical outcomes and that circCRIM1 was an independent factor for NPC survival, confirming the prognostic importance of circRNAs in NPC. However, little is known about the role of circRNAs in chemoresistance. We found that circCRIM1 knockdown enhanced docetaxel chemotherapy sensitivity in NPC cells. Our phase 3 study prospectively provided clinical evidence that an IC regimen containing docetaxel, cisplatin and fluorouracil significantly decreases distant metastasis and prolongs survival in LA-NPC patients when added to CCRT. Currently, the eligibility criteria are based mainly on TNM stage, which is not effective enough to identify ideal candidates for IC. Effective biomarkers predicting ideal candidates for IC remain under investigation. Our results demonstrated that the status of circCRIM1 expression combined with N stage could distinguish patients with different responses to docetaxel-containing IC. These findings may improve strategies to predict benefit from docetaxel-based IC treatment prior to patient exposure, which will avoid unnecessary toxicity and allow them to move on to alternative treatment options.

## Conclusion

In summary, our study identifies that circCRIM1 competitively sponges miR-422a to block the suppression effect of miR-422a on FOXQ1 and then contributes to NPC cell metastasis and docetaxel chemoresistance. We also highlight the clinical value of a circCRIM1-based prognostic model for NPC metastasis risk and docetaxel IC benefit. These data provide novel insight into understanding the progression and therapy resistance of NPC and potential predictive and therapeutic strategies for NPC patients.

## Methods

### Cell lines and cell culture

Human NPC cell lines (S18 and S26) were cultured in DMEM (Invitrogen, Grand Island, NY, USA) supplemented with 10% fetal bovine serum (FBS, Gibco, Grand Island, NY, USA). As reported previously [46], the highly metastatic subclone S18 and poorly metastatic subclone S26 were established using a limiting dilution method and confirmed using in vitro functional studies and in vivo animal experiments. The human NPC cell line HONE1 was cultured in RPMI 1640 (Invitrogen) supplemented with 10% FBS. These cell lines were incubated in a humidified chamber with 5% CO_2_ at 37 °C. All NPC cell lines, which had been authenticated, were graciously provided by Professor Mu-sheng Zeng (Sun Yat-Sen University Cancer Center). All the cells were authenticated using short-tandem repeat profiling, tested for mycoplasma contamination, and cultured for less than 2 months.

### Clinical specimens

A total of 16 freshly frozen normal nasopharyngeal epithelial tissues and 20 NPC biopsy tissues were collected from Sun Yat-Sen University Cancer Center (Guangzhou, China). For large cohort validation, we collected 218 formalin-fixed paraffin-embedded (FFPE) NPC specimens from Sun Yat-Sen University Cancer Center between January 2006 and December 2009. All patients were diagnosed with nonmetastatic NPC, and none received any antitumor therapy before biopsy. All patients were restaged according to the 8th edition of the American Joint Committee on Cancer (AJCC) staging manual. Detailed information about the clinicopathological characteristics of all patients was collected, and the median follow-up time was 69.45 months (range 2.6 to 115.87 months). In addition, all patients received radiotherapy with concurrent platinum-based chemotherapy alone or plus IC. Additional file 1: Table S4 shows the detailed clinicopathological characteristics.

### CircRNA isolation, library synthesis and RNA sequencing

TRIzol reagent (Invitrogen) was used to extract total RNA. The RNA amount and quality were measured using a NanoDropND-2000 spectrophotometer (Thermo Fisher Scientific, Rockford, IL, USA). Then, DNase I was used for DNA digestion, and ribosomal RNAs were depleted from the total RNA using a RiboMinus kit (Life Technologies, Thermo Fisher Scientific, Rockford, IL, USA), followed by RNase R treatment to enrich circRNAs. The remaining RNAs were fragmented and reverse-transcribed using random primers and reverse transcriptase. Second strand cDNA synthesis was completed using DNA polymerase I and RNase H. The Illumina series KAPA Library Preparation Kit (Kapa Biosystems) was used to perform end repair, dA-tailing, and adapter ligation. The cDNA was enriched by PCR amplification to create the final cDNA libraries and subjected to deep sequencing with an Illumina HiSeq™2000. After quality control on raw reads, HISAT software was utilized to map to the reference genome. Genes with |fold change| ≥ 2 and FDR < 0.05 were considered differentially expressed.

### RNA and gDNA extraction

TRIzol reagent (Invitrogen) was used to extract total RNA from NPC cell lines and freshly frozen NPC samples. A QIAGEN FFPE RNeasy kit (QIAGEN GmbH, Hilden, Germany) was used to extract total RNA from FFPE NPC samples. For RNase R treatment, total RNA (10 μg) was incubated for 45 min at 37 °C with or without 2 U/μg RNase R (Epicentre, WI, USA). gDNA was extracted using a TIANamp Genomic DNA Kit (TIANGEN Biotech, Beijing, China). Cytoplasmic and nuclear fractions of S18 cells were extracted using NE-PER Nuclear and Cytoplasmic Extraction Reagents (Thermo Fisher Scientific) according to the manufacturer’s instructions.

### Quantitative real-time PCR

Reverse transcription was performed using reverse transcriptase (Promega, Madison, WI, USA) and random primers (Promega) for circRNA and mRNA or Bulge-Loop miRNA-specific RT-primers (RiboBio, Guangzhou, China) for miRNA. Real-time PCR reactions were performed with SYBR Green qPCR Super Mix-UDG reagent (Invitrogen) on a CFX96 Touch sequence detection system (Bio-Rad, Hercules, CA, USA). For cell lines and freshly frozen specimens, GAPDH and U6 were used as internal controls for circRNA/mRNA and miRNA, respectively. For FFPE samples, β-actin was used as the normalization control for circRNA. The relative expression levels were calculated using the 2^-ΔΔCT^ or 2^-ΔCT^ method. Specific primers for circRNA and mRNA are shown in Additional file 1: Table S7.

### Oligonucleotide and plasmid transfection

A miR-422a mimic and inhibitor and specific siRNA oligonucleotides that targeted circCRIM1 or FOXQ1 were purchased from RiboBio. To overexpress circCRIM1, full length circCRIM1 was cloned into a modified LV003 lentiviral vector, which contained a front circular frame and a back circular frame, while an empty vector served as a control. We mutated the circCRIM1 expressing plasmid (circCRIM1-si-mut) at the back-spliced junction by reversing 5’end of CRIM1 mRNA sequence “ATGAGAA” into “AAGAGTA” and 3’end of CRIM1 mRNA sequence “CAACAAG” into “GAACAAC”. The circCRIM1-si-mut plasmid was constructed with the mutant back splice junction of “GAACAACAAGAGTA”. All vectors were verified by sequencing. Cell transfections were conducted with Lipofectamine 3000 (Invitrogen) in accordance with the manufacturer’s protocols. The cells were harvested for assays 48 h after transfection. The sequences of siRNAs are listed in Additional file 1: Table S7.

### Transwell migration and invasion assays

Cell migration and invasion abilities were detected with Transwell chambers (8-μm pores; Corning, Corning, NY, USA) that were coated without or with Matrigel (BD Biosciences, NJ, USA). NPC cells (5 × 10^4^ or 1 × 10^5^) suspended in serum-free medium were plated in the upper chambers. Medium supplemented with 10% FBS was placed in the lower chambers. After incubation for 12–24 h, the migrated or invaded cells were fixed, stained and counted using an inverted microscope.

### Immunofluorescence staining (IF)

NPC cells grown on cell climbing pieces were rinsed twice with PBS and fixed with 4% paraformaldehyde for 15 min. After washing three times with PBS, the cells were permeabilized with 0.1% Triton-100 in PBS for 10 min on ice. The cells were then washed twice with PBS, blocked with 5% BSA in PBST for 30 min at 37 °C and incubated with primary anti-E-cadherin (1:100; BD Biosciences, 610,181) and anti-Vimentin (1:250; Proteintech, Rosemont, USA, 10366–1-AP) antibodies overnight at 4 °C. After washing with PBST, the cells were incubated with the corresponding secondary antibody for 45 min at room temperature. The cells were then stained with Hoechst (Invitrogen) for nuclear staining. Fluorescent images were acquired using a confocal laser-scanning microscope (Olympus FV1000, Tokyo, Japan).

### Western blotting assay

Proteins were extracted with RIPA lysis buffer (Beyotime Biotechnology, Shanghai, China) containing EDTA-free Protease Inhibitor Cocktail (Roche, Basel, Switzerland) and determined using a BCA protein assay kit (Thermo Fisher Scientific). Protein extracts were separated via 4–20% SDS-PAGE and transferred to polyvinylidene fluoride membranes (Merck Millipore, Billerica, MA, USA). The membranes were blocked in 5% nonfat milk and incubated with primary antibodies targeting FOXQ1 (1:500, Abcam, Cambridge, UK, ab51340), E-cadherin (1:1000, Cell Signaling Technology, Danvers, MA, USA, 3195S), N-cadherin (1:500, BD Biosciences, 610,921), Vimentin (1:1000, Proteintech, 10,366–1-AP), and α-tubulin (1:1000, Proteintech, 66,031–1-Ig) overnight at 4 °C. Secondary antibody incubation was performed using horseradish peroxidase-conjugated antibodies (anti-mouse or anti-rabbit; 1:5000; Proteintech) at room temperature for 1 h. Finally, the target protein bands were detected using an ECL detection system (Thermo Fisher Scientific), and quantified values were reported after normalizing to the loading controls. Full unedited western blotting gels were shown in Additional file 1: Figure S11.

### RNA immunoprecipitation (RIP)

RIP was conducted with a Magna RIP kit (Millipore) following the manufacturer’s instructions. S18 and HONE1 cells were harvested and lysed in complete RIP lysis buffer. Then, cell lysates were incubated with magnetic beads conjugated with anti-Argonaute2 (AGO2, Abcam, ab57113) or a negative control IgG antibody (Millipore) on a rotator overnight at 4 °C. The beads were washed using washing buffer. Then, immunoprecipitated RNA and protein were purified and enriched to detect the target RNAs and AGO2 by RT-qPCR or Western blotting.

### Dual luciferase reporter assay

Full length circCRIM1, FOXQ1–3′ UTR and their corresponding mutant versions with mutant miR-422a binding sites were synthesized and cloned into the luciferase reporter vector psiCHECK-2. All these plasmids were validated by sequencing. S18 and HONE1 cells (5 × 10^4^) were seeded into 24-well plates and cotransfected with corresponding plasmids and microRNA mimics using Lipofectamine 3000. After 48 h incubation, the cells were lysed, and the relative luciferase activity was exanimated using a Dual Luciferase Assay Kit (Promega) in accordance with the manufacturer’s protocol.

### Pull-down assay with biotinylated miRNA

The biotinylated 3′ end of miR-422-a mimic or control RNA (RiboBio) was transfected into S18 and HONE1 cells at a final concentration of 100 nM. Two days after transfection, whole cell lysates were harvested. The biotinylated RNA complex was pulled down by incubating the cell lysates with streptavidin-coated magnetic beads at 4 °C on the rotator overnight. TRIzol LS reagent (Thermo Fisher Scientific) was used to extract RNA from the input and pull-down beads. The abundance of circCRIM1 in the bound fraction was evaluated by RT-qPCR analysis.

### Fluorescence in situ hybridization (FISH)

FISH assays were performed to observe the location of circCRIM1 and miR-422a in NPC cells. Briefly, after prehybridization at 55 °C for 2 h, cell climbing pieces were hybridized with a specific Cy3-labeled circCRIM1 probe (Cy3–5′-CCAGTTCTCATCTTGTTGGCAAAGTA-3′-Cy3) and FITC-labeled miR-422a probe (FITC-5′-GCCTTCTGACCCTAAGTCCAGT-3′-FITC) (Geneseed, Guangzhou, China) at 37 °C overnight and dyed with 4′, 6-diamidino-2-phenylindole (DAPI). Slides were photographed with a fluorescence microscope (Leica, Wetzlar, Germany).

### Gene set enrichment analysis (GSEA)

Gene expression profiles of 31 NPC specimens (GSE12452) were used to conduct GSEA to identify gene signatures between groups with high and low FOXQ1 expression. C2 collection of chemical and genetic perturbations (*n* = 3409 gene sets) from the Molecular Signatures Database (http://www.broad.mit.edu/gsea/msigdb/index.jsp) was used for the enrichment analysis. GSEA results are shown using normalized enrichment scores, and a threshold of *P* < 0.05 and FDR ≤ 0.25 was used to select significant items.

### CCK8 and colony formation assays

For CCK8 assays, S18 and HONE1 cells were seeded into 96-well cell culture plates (2000 cells in 100 μl medium per well). After 12 h, docetaxel was added to the cell culture medium. After a 2-day treatment, 10 μl CCK8 solution (Dojindo, Shanghai, China) was added into each well of the plate. The plate was incubated for 2 h at 37 °C in the cell incubator and then measured on a spectrophotometric plate reader (BioTek ELX800, Bio-Rad) at OD450 nm. For colony formation assays, 1000 cells in 2 ml medium were seeded in 6-well plates. After 12 h, docetaxel was added to the cell culture medium. After 1–2 weeks, the colonies were washed, fixed, stained and counted.

### In vivo xenograft tumor models

Six-week-old female BALB/c nude mice were purchased from Beijing Vital River Lab Animal Technology Co., LTD. For in vivo lymph node metastasis, S18 cells (2 × 10^5^ cells in 30 μl serum-free DMEM medium containing 20% Matrigel) were injected into the mouse footpads (*n* = 8 per group). After 2 weeks, cholesterol-conjugated circCRIM1 siRNA or scrambled control siRNA (siSCR) was injected into the footpad tumors twice a week for 2 weeks. The footpad tumors and inguinal lymph nodes were excised for analysis. For lung metastasis model construction, 1 × 10^6^ S18 cells were injected into the mice via their tail vein. Two weeks later, cholesterol-conjugated circCRIM1/FOXQ1 siRNA or siSCR was administered via tail vein injection (*n* = 5 per group) at a dose of 5 nM, twice per week for 5 weeks. The mice were sacrificed, and their lung tissues were dissected. All the dissected tissue samples were paraffin-embedded, sectioned and stained with H&E. Additionally, IHC staining was performed on the inguinal lymph nodes using an anti-pan-cytokeratin antibody (Thermo Fisher Scientific). For the lung metastasis model following docetaxel treatment, S18 cells were injected into the tail veins of mice. Two weeks later, cholesterol-conjugated circCRIM1-siRNA at a dose of 5 nM was administered to the mice via tail vein injection twice a week for 5 weeks. Two weeks after first si-circCRIM1 administration, docetaxel at a dose of 1.5 mg/kg were delivered via intraperitoneal injection, once per week for 3 weeks.

### Immunohistochemical staining (IHC)

IHC was performed on xenograft mouse tissues. All sections were deparaffinized with xylene and rehydrated with a gradient of ethanol to distilled water. After treatment with citrate buffer, the tissue sections were preincubated with hydrogen peroxide and blocked with goat serum (Beyotime). The sections were then incubated with a primary antibody against FOXQ1 (1:200; Abcam, ab51340), labeled with an avidin-biotin peroxidase complex (Dako, Glostrup, Denmark) followed by diaminobenzidine development (Sigma-Aldrich, Ronkonkoma, NY, USA). Finally, sections were counterstained with hematoxylin. The stained results were reviewed and scored as described in previous literature.

### Statistical analysis

SPSS 22.0 software (IBM, Armonk, NY, USA) was used for all statistical analyses, and a *P* value < 0.05 was considered significant. All data are from no less than three independent experiments. A two-tailed Student’s *t*-test was used to compare continuous variables. χ^2^ or Fisher’s exact test was used to compare categorical variables. Receiver operating characteristic (ROC) curve analysis was performed to determine the optimal cutoff value for the classification of high or low circCRIM1 expression. The Kaplan-Meier method and log-rank test were used to construct survival curves and compare differences. Multivariate analysis was used to determine independent prognostic factors using a Cox proportional hazards regression model.

## Supplementary information

**Additional file 1: Figure S1.** Schematic map of circRNA isoforms arising from CRIM1 genome and relative circCRIM1 expression in NPC cells and tissues. **Figure S2.** Knockdown and overexpression efficiency of circCRIM1 or miR-422a in NPC cells. **Figure S3–4.** Multiple sequence alignment of two independent siRNAs targeting circCRIM1 and 15 circRNA isoforms derived from *CRIM1* gene in S18 cells. **Figure S5.** Transwell assays for the rescue effects of circCRIM1 with a mutant backsplice junction of circCRIM1. **Figure S6.** CircCRIM1 induces EMT in NPC cells. **Figure S7.** FOXQ1 is a downstream target of miR-422a and circCRIM1 involved in metastasis. **Figure S8.** FOXQ1 downregulation inhibited NPC cell metastasis in vivo. **Figure S9.** circCRIM1 downregulation enhances the docetaxel efficacy on NPC cell metastasis in vivo. **Figure S10.** Patients in the combined intermediate and high risk groups did not benefit from docetaxel-containing induction chemotherapy. **Figure S11.** Full unedited Western blotting gels for all figures. **Table S1:** List of 15 circRNA isoforms derived from *CRIM1* gene in S18 cells. **Table S2.** Downregulated miRNAs according to miRNA microarray. **Table S3.** Putative miRNAs predicted to bind circCRIM1 by miRanda and RNAhybrid algorithms. **Table S4.** Putative miRNAs predicted to bind circCRIM1 by StarBase algorithms. **Table S5.** Clinical characteristics of NPC patients according to high and low circCRIM1 expression. **Table S6.** Univariate and multivariable Cox regression analysis of circCRIM1 expression level and survival in NPC patients. **Table S7.** Primers and RNA sequences used in this study.

**Additional file 2. **List of circRNA isoforms derived from the *CRIM1* gene based on circBase database.

## Data Availability

The microarray and circular RNA-Sequencing datasets used in this paper have been deposited at Gene Expression Omnibus (http://www.ncbi.nlm.nih.gov/geo/) under the series accession number GSE12452 (microarray data of NPC tissues), GSE137312 (microarray data of NPC cell lines) and GSE137543 (circular RNA-Sequencing data of NPC cell lines). CircRNA isoforms derived from *CRIM1* gene are identified based on circBase database (http://www.circbase.org/). The authenticity of this article has been validated by uploading the key raw data onto the Research Data Deposit public platform (http://www.researchdata.org.cn) No. RDDB2020000800.

## Abbreviations

AGO2: Argonaute 2
ceRNAs: Competitive endogenous RNAs
CI: Confidence interval
circRNAs: Circular RNAs
DFS: Disease-free survival
DMFS: Distant metastasis-free survival
FFPE: Formalin-fixed paraffin-embedded
FOXQ1: Forkhead box Q1
gDNA: Genomic DNA
GSEA: Gene set enrichment analysis
H&E: Hematoxylin and eosin
HR: Hazard ratio
IC: Induction chemotherapy
IHC: Immunohistochemical staining
LA-NPC: Locoregionally advanced nasopharyngeal carcinoma
ncRNA: Noncoding RNA
NPC: Nasopharyngeal carcinoma
OS: Overall survival
qRT-PCR: Quantitative real-time PCR
RIP: RNA immunoprecipitation
RISC: Silencing complex
ROC: Receiver operating characteristic
siRNAs: Short interfering RNAs
siSCR: Scrambled control siRNA
TNM: Tumor-node-metastasis
