# Supplementary material for: Circular RNA CRIM1 functions as a ceRNA to promote nasopharyngeal carcinoma metastasis and docetaxel chemoresistance through upregulating FOXQ1
Source: Mol Cancer. 2020 Feb 15;19:33. doi: 10.1186/s12943-020-01149-x (PMC7023763; doi:10.1186/s12943-020-01149-x)
Supplement: Supplementary file 1 — Additional file 1: Figure S1. Schematic map of circRNA isoforms arising from CRIM1 genome and relative circCRIM1 expression in NPC cells and tissues. Figure S2. Knockdown and overexpression efficiency of circCRIM1 or miR-422a in NPC cells. Figure S3–4. Multiple sequence alignment of two independent siRNAs targeting circCRIM1 and 15 circRNA isoforms derived from CRIM1 gene in S18 cells. Figure S5. Transwell assays for the rescue effects of circCRIM1 with a mutant backsplice junction of circCRIM1. Figure S6. CircCRIM1 induces EMT in NPC cells. Figure S7. FOXQ1 is a downstream target of miR-422a and circCRIM1 involved in metastasis. Figure S8. FOXQ1 downregulation inhibited NPC cell metastasis in vivo. Figure S9. circCRIM1 downregulation enhances the docetaxel efficacy on NPC cell metastasis in vivo. Figure S10. Patients in the combined intermediate and high risk groups did not benefit from docetaxel-containing induction chemotherapy. Figure S11. Full unedited Western blotting gels for all figures. Table S1: List of 15 circRNA isoforms derived from CRIM1 gene in S18 cells. Table S2. Downregulated miRNAs according to miRNA microarray. Table S3. Putative miRNAs predicted to bind circCRIM1 by miRanda and RNAhybrid algorithms. Table S4. Putative miRNAs predicted to bind circCRIM1 by StarBase algorithms. Table S5. Clinical characteristics of NPC patients according to high and low circCRIM1 expression. Table S6. Univariate and multivariable Cox regression analysis of circCRIM1 expression level and survival in NPC patients. Table S7. Primers and RNA sequences used in this study. [file 12943_2020_1149_MOESM1_ESM.docx]

**Supplementary Figures and Tables**


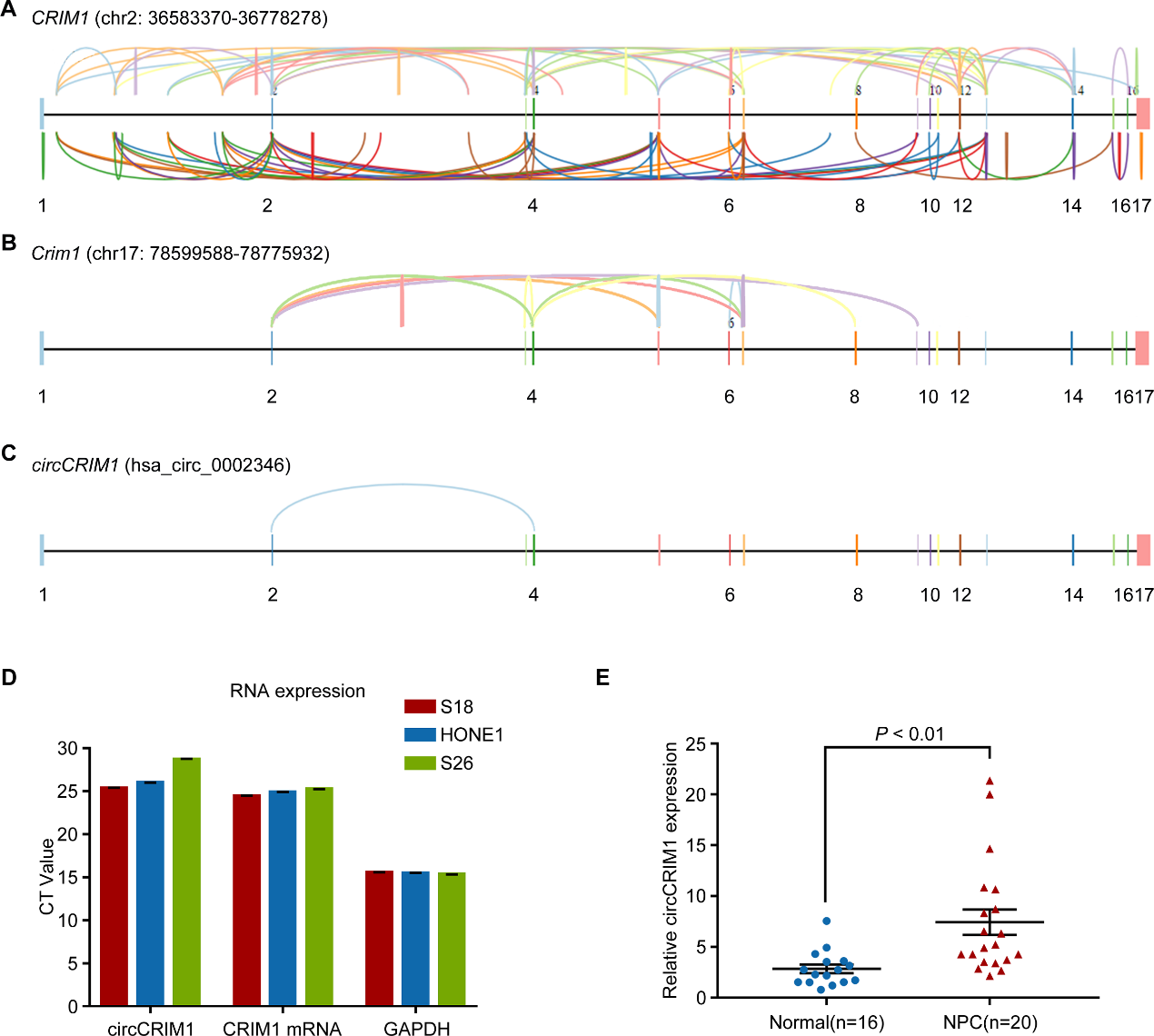


**Figure S1. Schematic map of circRNA isoforms arising from *CRIM1* genome and relative circCRIM1 expression in NPC cells and tissues. a-c** Schematic map of circRNA isoforms arising from *CRIM1* gene referenced to *Homo Sapiens* (**a**) and *Mus Musculus* (**b**) species genome. **c** hsa_circ_0002346 isoform. Linear CRIM1 structures are displayed with different colored rectangles for exons and black lines for introns, while circRNAs derived from *CRIM1* gene are shown as colorful curves. **d** qRT-PCR analyses of the expression of circCRIM1, CRIM1 mRNA and GAPDH in NPC cell lines. Y-axis is the raw CT value. **e** Relative circCRIM1 expression in normal (*n* = 16) and nasopharyngeal carcinoma (*n* = 20) tissues. β-actin was used a normalization control. Mean ± S.D. Student’s *t*-tests.

**
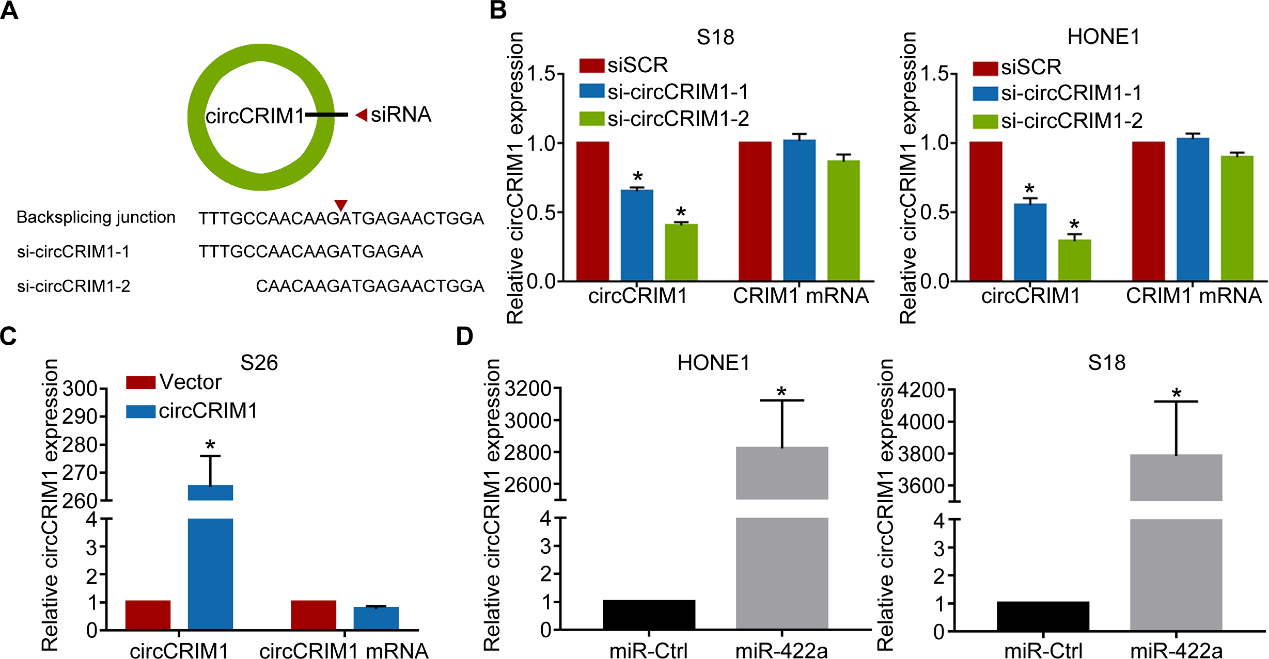
**

**Figure S2. Knockdown and Overexpression efficiency of circCRIM1 or miR-422a in NPC cells. a** Schematic representation of the siRNA specific sites to the back-splice junction of circCRIM1. **b-c** Relative circCRIM1 and CRIM1 mRNA expression with or without circCRIM1 downregulation (**b**) or overexpression (**c**). **d** Relative miR-422a expression after transfection of miR-422a mimics or miRNA control (miR-Ctrl). Mean (*n* = 3) ± S.D. Student’s *t*-tests; **P* < 0.05.


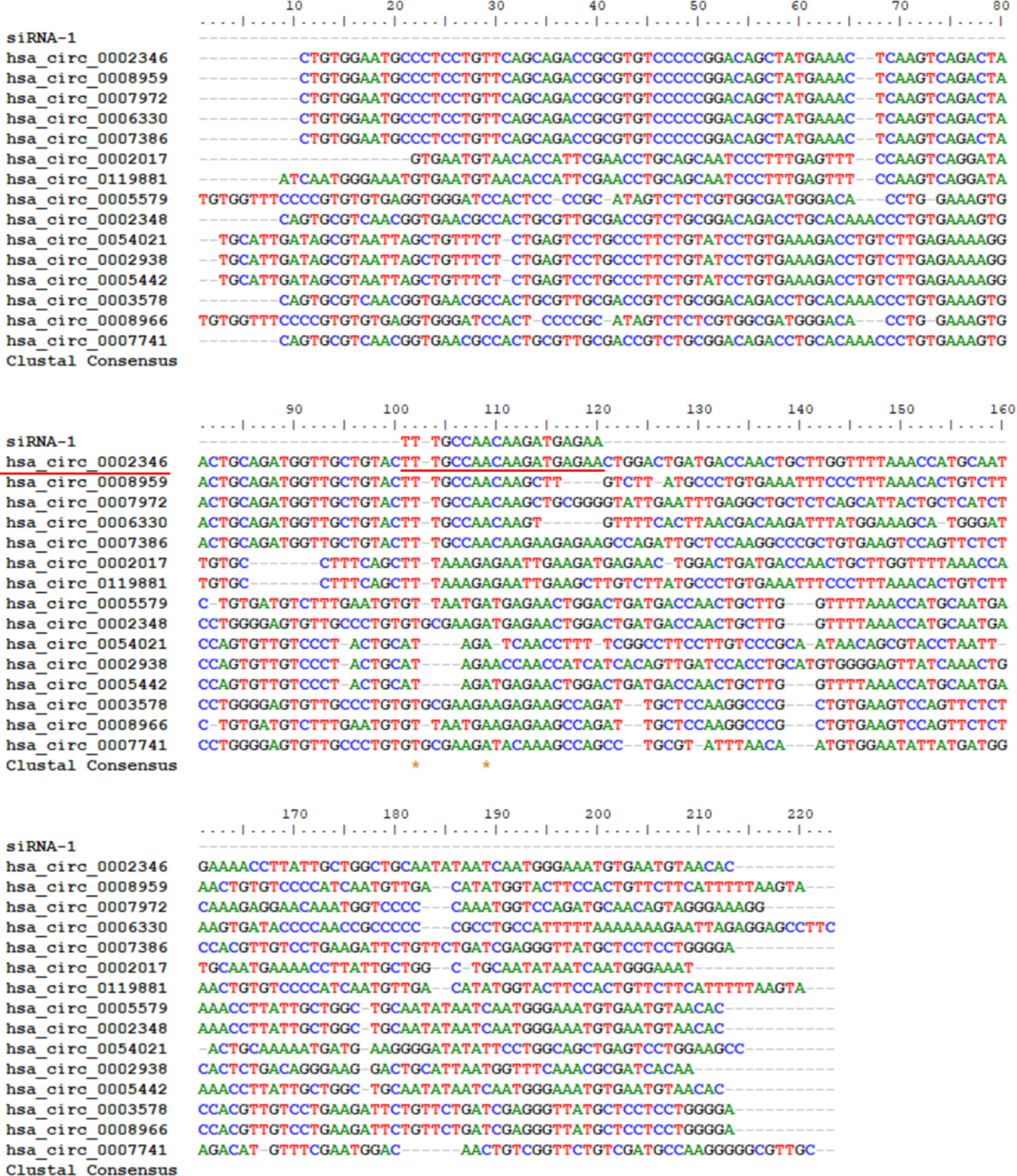


**Figure S3.** **Multiple sequence alignment of siRNA-1** **targeting circCRIM1 and 15 circRNA isoforms derived from *CRIM1* gene in S18 cells.** Sequences of siRNA-1 targeting circCRIM1 and one hundred bases before and after back-spliced junction sites of each isoforms were submitted to make sequence alignment analyses. Only circCRIM1 has completely complementary sequences to siRNA-1.


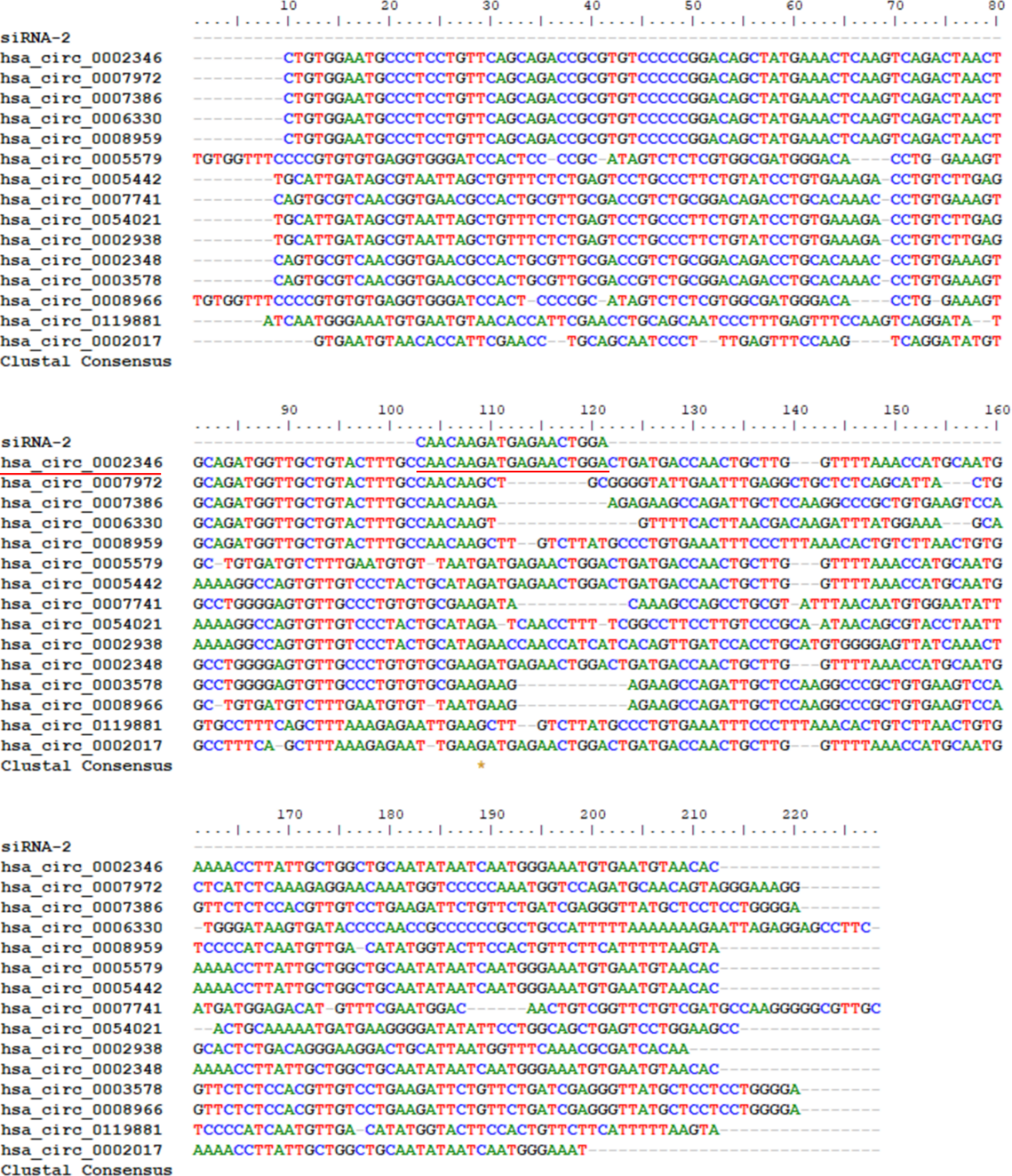


**Figure S4. Multiple sequence alignment of siRNA-2 targeting circCRIM1 and 15 circRNA isoforms derived from *CRIM1* gene in S18 cells.** Sequences of siRNA-2 targeting circCRIM1 and one hundred bases before and after back-spliced junction sites of each isoforms were submitted to make sequence alignment analyses. Only circCRIM1 has completely complementary sequences to siRNA-2.

**
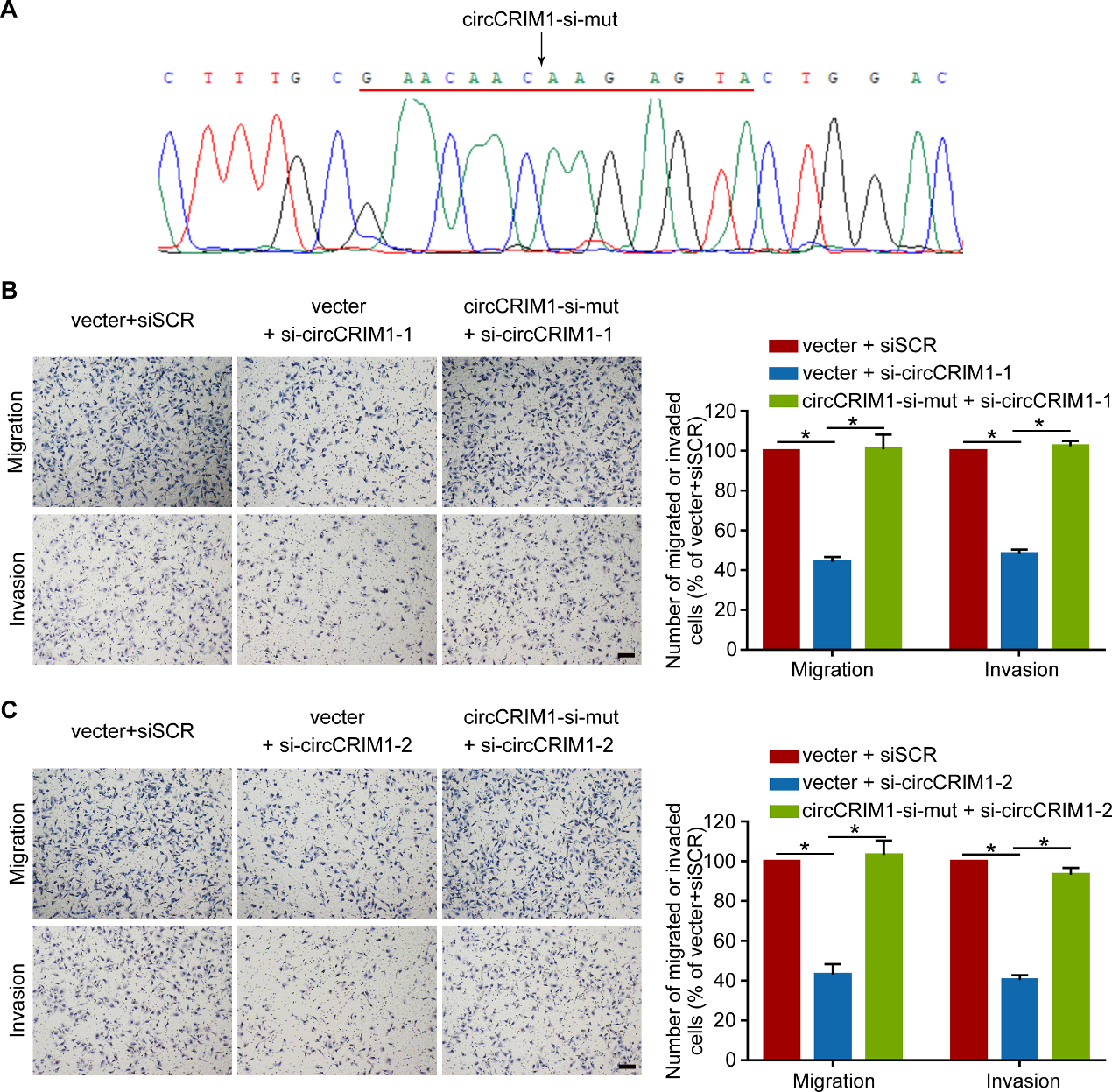
**

**Figure S5. Transwell assays for the rescue effects of circCRIM1 with a mutant backsplice junction of circCRIM1. a** Sanger sequencing for the mutant back-spliced junction of circCRIM1. The circCRIM1-si-mut plasmid was constructed with the mutant back splice junction of “GAACAACAAGAGTA”. **b-c** Representative and quantified results of the Transwell migration and invasion assays in S18 cells cotransfected with either si-circCRIM1-1 (**b**) or si-circCRIM1-2 (**c**) or scrambled control and circCRIM1-si-mut plasmid or vector. Mean (*n* = 3) ± S.D. Student’s *t*-tests; **P* < 0.05. Scale bar, 100 µm.

**
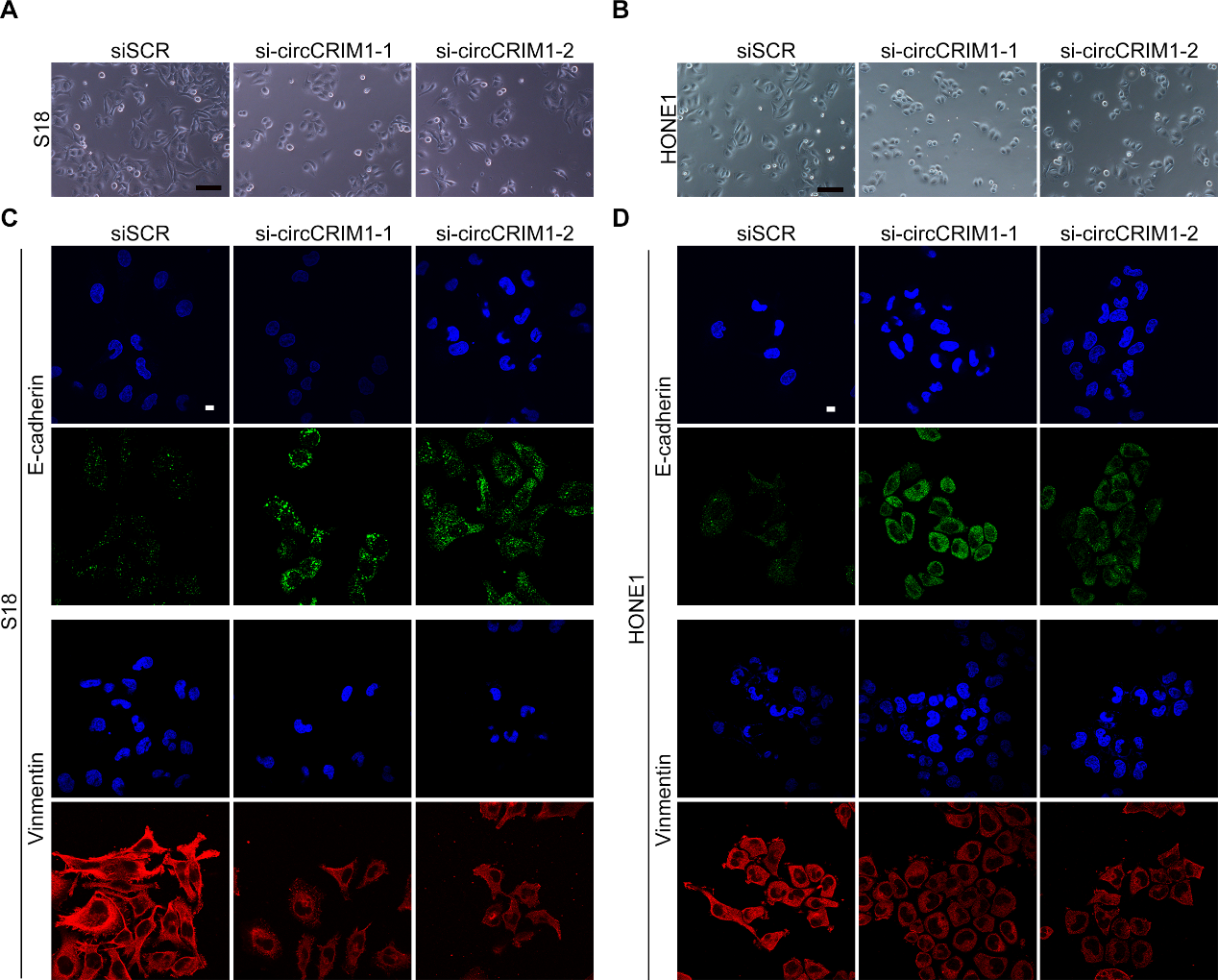
**

**Figure S6. CircCRIM1 induces EMT in NPC cells. a-b** Phase contrast images of S18 (**a**) and HONE1 (**b**) cells transfected with circCRIM1 siRNA or scramble control (siSCR). Scale bar, 100um. **c-d** Original single-channel immunofluorescence images for E-cadherin and Vimentin expression in S18(**c**) and HONE1(**d**) cells transfected with circCRIM1 siRNA or siSCR. Scale bar, 10um.


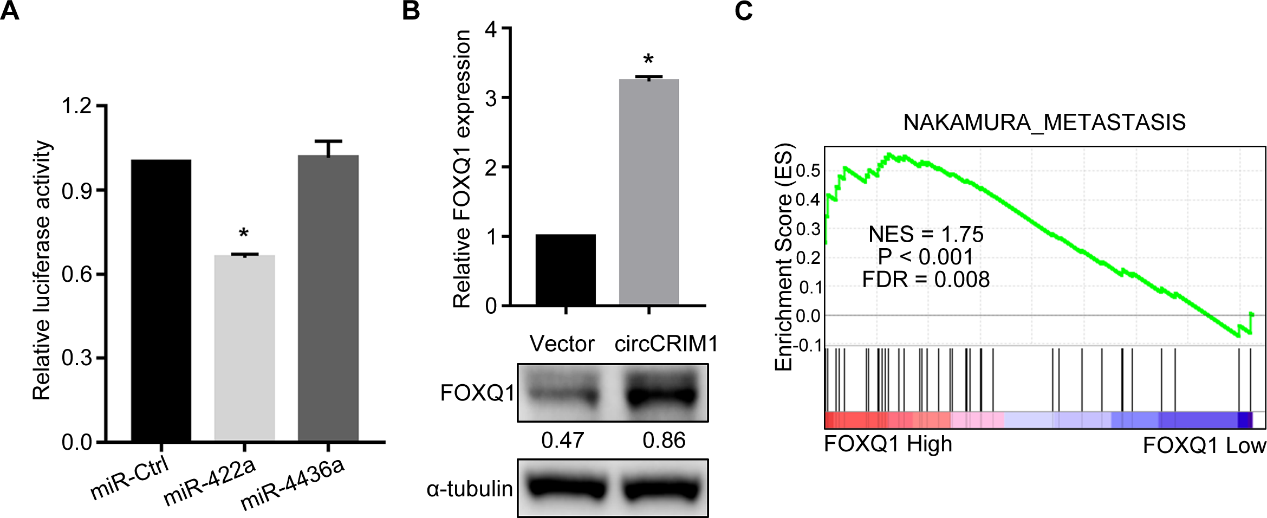


**Figure S7.** **FOXQ1 is a downstream target of miR-422a and circCRIM1 involved in metastasis. a** Luciferase activity of wild type circCRIM1 in HONE1 cell after co-transfection with miR-Ctrl, miR-422a or miR-4436a mimics. Mean (*n* = 3) ± S.D. Student’s *t*-tests; **P* < 0.05. **b** qRT-PCR and Western blotting analysis of FOXQ1 expression in NPC cells transfected with and without plasmids overexpressing circCRIM1. Mean (*n* = 3) ± S.D. Student’s *t*-test; **P* < 0.05. **c** Metastasis-related biological functions enriched in response to high FOXQ1 expression in NPC patients.


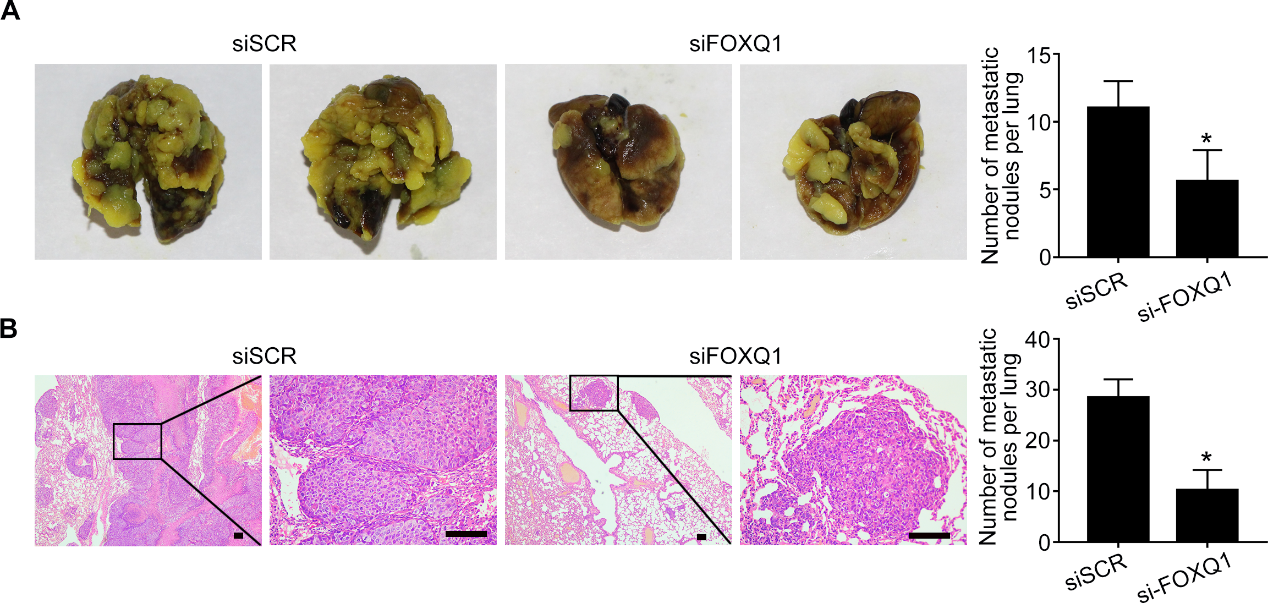


**Figure S8. FOXQ1 downregulation inhibited NPC cell metastasis *in vivo*.** S18 cells were intravenously injected via the tail vein into nude mice. Two weeks later, cholesterol-conjugated FOXQ1-siRNA at a dose of 5 nM was administered to the mice via tail vein injection twice a week for 5 weeks. Representative images and quantification of macroscopic (**a**) and microscopic metastatic nodules (**b**) in the lungs of mice. Mean (*n* = 5) ± s.d. Student’s *t*-test, * *P* < 0.05. Scale bar, 100 μm.


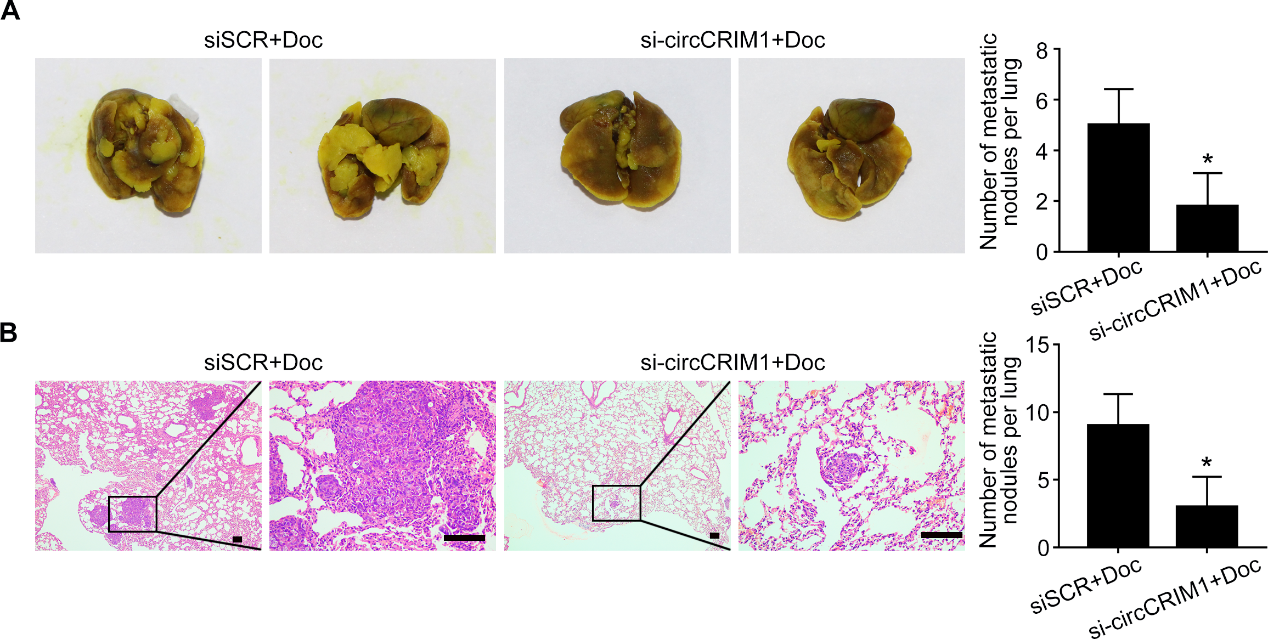


**Figure S9. circCRIM1 downregulation enhances the docetaxel efficacy on NPC cell metastasis *in vivo*.** S18 cells were injected into the tail veins of mice. Two weeks later, cholesterol-conjugated circCRIM1-siRNA at a dose of 5 nM was administered to the mice via tail vein injection twice a week for 5 weeks. Two weeks after first si-circCRIM1 administration, docetaxel at a dose of 1.5 mg/kg were delivered via intraperitoneal injection, once per week for 3 weeks. Representative images and quantification of macroscopic (**a**) and microscopic metastatic nodules (**b**) in the lungs of mice. Mean (*n* = 5) ± s.d. Student’s *t*-test, * *P* < 0.05. Scale bar, 100 μm.


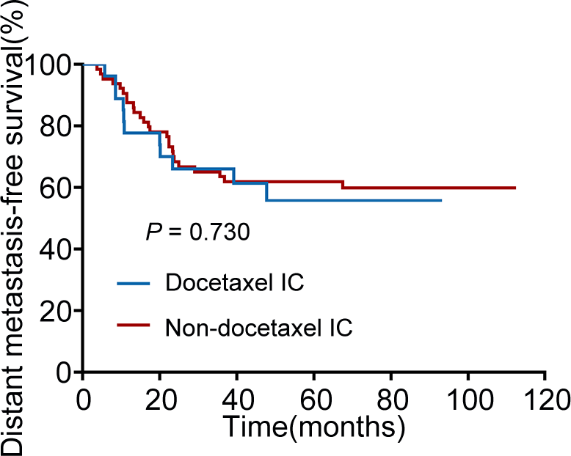


**Figure S10. Patients in combined intermediate and high risk groups did not benefit from docetaxel-containing** **induction chemotherapy.** DMFS in intermediate- and high-risk patients received induction chemotherapy with and without docetaxel. *P* values were determined using the log-rank test.


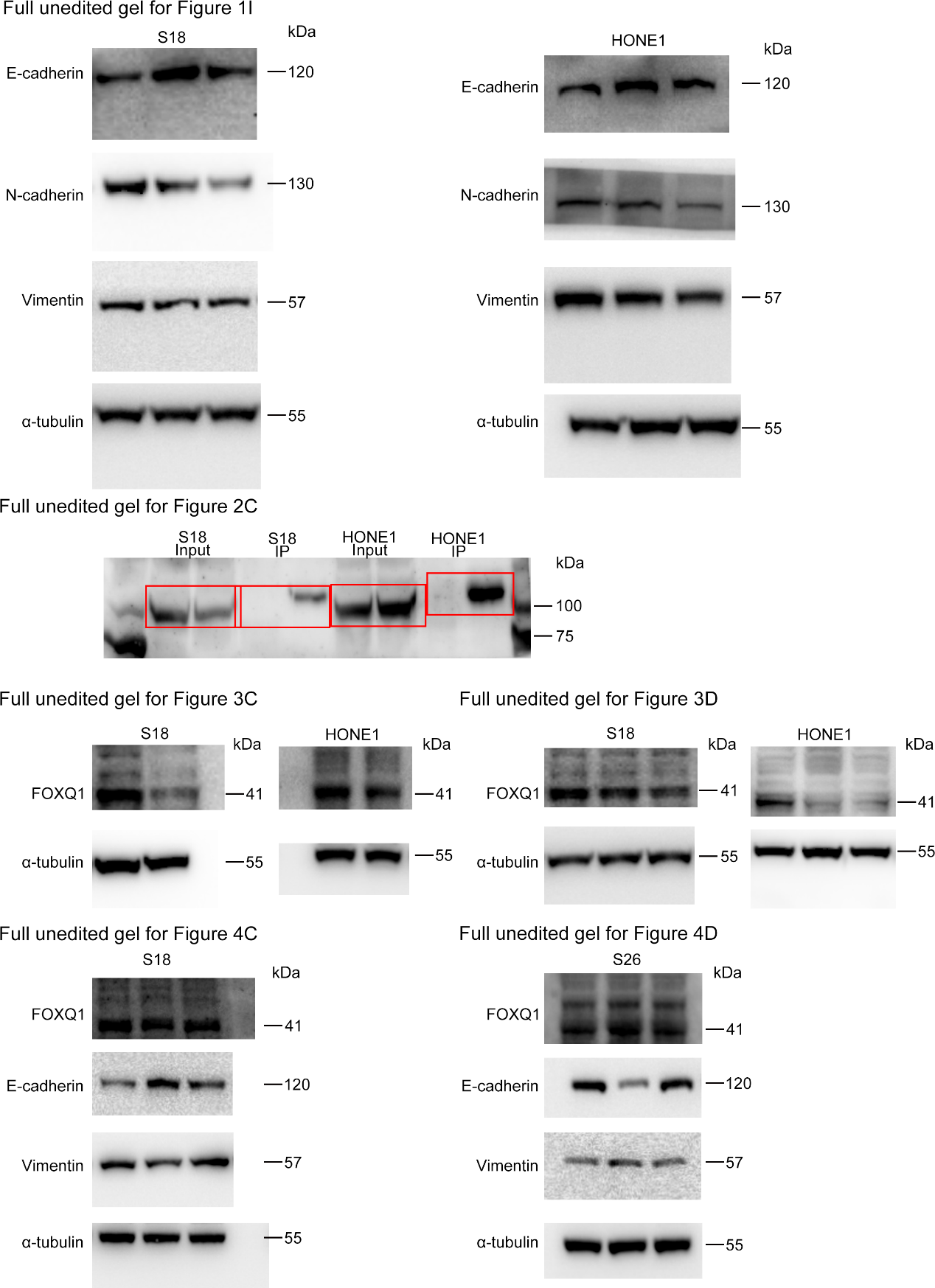


**Figure S11. Full unedited western blotting gels for all figures.**

**Table S1: List of 15 circRNA isoforms derived from *CRIM1* gene in S18 cells.**

| CircRNA ID | Chr | start | end | strand | Gene name |
| --- | --- | --- | --- | --- | --- |
| hsa_circ_0005579 | chr2 | 36623756 | 36691798 | + | CRIM1 |
| hsa_circ_0002017 | chr2 | 36623756 | 36623930 | + | CRIM1 |
| hsa_circ_0002346 | chr2 | 36623756 | 36669878 | + | CRIM1 |
| hsa_circ_0005442 | chr2 | 36623756 | 36749456 | + | CRIM1 |
| hsa_circ_0003578 | chr2 | 36668400 | 36706837 | + | CRIM1 |
| hsa_circ_0054021 | chr2 | 36749234 | 36749456 | + | CRIM1 |
| hsa_circ_0006330 | chr2 | 36586046 | 36669878 | + | CRIM1 |
| hsa_circ_0008966 | chr2 | 36668400 | 36691798 | + | CRIM1 |
| hsa_circ_0008959 | chr2 | 36615114 | 36669878 | + | CRIM1 |
| hsa_circ_0002938 | chr2 | 36726361 | 36749456 | + | CRIM1 |
| hsa_circ_0002348 | chr2 | 36623756 | 36706837 | + | CRIM1 |
| Hsa_circ_0007741 | chr2 | 36704031 | 36706837 | + | CRIM1 |
| hsa_circ_0119881 | chr2 | 36615114 | 36623930 | + | CRIM1 |
| hsa_circ_0007972 | chr2 | 36605499 | 36669878 | + | CRIM1 |

**Table S2.** **Downregulated miRNAs according to miRNA microarray**

| downregulated miRNAs according to miRNA microarray | | | |
| --- | --- | --- | --- |
| hsa-miR-1254 | hsa-miR-345-3p | hsa-miR-4448 | hsa-miR-509-5p |
| hsa-miR-1287 | hsa-miR-34c-3p | hsa-miR-4508 | hsa-miR-512-3p |
| hsa-miR-1295a | hsa-miR-3605-5p | hsa-miR-4514 | hsa-miR-513c-3p |
| hsa-miR-133a | hsa-miR-3607-5p | hsa-miR-4522 | hsa-miR-514b-5p |
| hsa-miR-139-3p | hsa-miR-3614-5p | hsa-miR-4531 | hsa-miR-516b-5p |
| hsa-miR-141-5p | hsa-miR-3622a-5p | hsa-miR-4638-5p | hsa-miR-5196-5p |
| hsa-miR-193a-5p | hsa-miR-3675-3p | hsa-miR-4642 | hsa-miR-542-5p |
| hsa-miR-200a-3p | hsa-miR-3679-3p | hsa-miR-4652-3p | hsa-miR-550b-2-5p |
| hsa-miR-200b-3p | hsa-miR-378d | hsa-miR-4666b | hsa-miR-5696 |
| hsa-miR-200c-5p | hsa-miR-378f | hsa-miR-4675 | hsa-miR-614 |
| hsa-miR-202-3p | hsa-miR-3935 | hsa-miR-4685-3p | hsa-miR-623 |
| hsa-miR-205-3p | hsa-miR-3937 | hsa-miR-4707-5p | hsa-miR-628-3p |
| hsa-miR-205-5p | hsa-miR-3972 | hsa-miR-4725-3p | hsa-miR-648 |
| hsa-miR-2277-3p | hsa-miR-422a | hsa-miR-4728-3p | hsa-miR-6500-5p |
| hsa-miR-299-3p | hsa-miR-4253 | hsa-miR-4731-3p | hsa-miR-6507-3p |
| hsa-miR-3131 | hsa-miR-4280 | hsa-miR-4732-5p | hsa-miR-6509-5p |
| hsa-miR-3147 | hsa-miR-429 | hsa-miR-4740-5p | hsa-miR-6511a-5p |
| hsa-miR-3150b-5p | hsa-miR-4304 | hsa-miR-4749-5p | hsa-miR-664a-5p |
| hsa-miR-3161 | hsa-miR-4314 | hsa-miR-4753-5p | hsa-miR-668 |
| hsa-miR-3173-3p | hsa-miR-4418 | hsa-miR-4769-5p | hsa-miR-671-3p |
| hsa-miR-3180-3p | hsa-miR-4419b | hsa-miR-4773 | hsa-miR-6720-3p |
| hsa-miR-3190-3p | hsa-miR-4429 | hsa-miR-4792 | hsa-miR-758-5p |
| hsa-miR-3197 | hsa-miR-4436a | hsa-miR-492 | hsa-miR-760 |
| hsa-miR-3200-5p | hsa-miR-4441 | hsa-miR-5003-5p |  |
| hsa-miR-338-3p | hsa-miR-4444 | hsa-miR-508-5p |  |

**Table S3.** **Putative miRNAs predicted to bind circCRIM1 by interacting miRanda and RNAhybrid algorithms**

| miRNAs predicted by miRanda & RNAhybrid algorithms | | | |
| --- | --- | --- | --- |
| hsa-miR-3127-5p | hsa-miR-93-3p | hsa-miR-6511b-5p | hsa-miR-4505 |
| hsa-miR-17-3p | hsa-miR-216a-3p | hsa-miR-6736-5p | hsa-miR-4515 |
| hsa-miR-1180-3p | hsa-miR-146a-5p | hsa-miR-6742-5p | hsa-miR-4525 |
| hsa-miR-1226-5p | hsa-miR-149-5p | hsa-miR-6749-5p | hsa-miR-3973 |
| hsa-miR-1231 | hsa-miR-34b-5p | hsa-miR-6767-5p | hsa-miR-4665-5p |
| hsa-miR-1205 | hsa-miR-378a-3p | hsa-miR-4436a | hsa-miR-5787 |
| hsa-miR-1910-5p | hsa-miR-665 | hsa-miR-6861-5p | hsa-miR-4692 |
| hsa-miR-885-3p | hsa-miR-328-5p | hsa-miR-5585-3p | hsa-miR-5589-5p |
| hsa-miR-3126-5p | hsa-miR-378h | hsa-miR-6885-5p | hsa-miR-4734 |
| hsa-miR-889-5p | hsa-miR-431-5p | hsa-miR-6892-5p | hsa-miR-4761-3p |
| hsa-miR-3166 | hsa-miR-486-3p | hsa-miR-7110-3p | hsa-miR-4779 |
| hsa-miR-3615 | hsa-miR-487b-5p | hsa-miR-7156-3p | hsa-miR-5001-5p |
| hsa-miR-3616-3p | hsa-miR-557 | hsa-miR-7157-5p | hsa-miR-5009-5p |
| hsa-miR-3681-3p | hsa-miR-567 | hsa-miR-7703 | hsa-miR-5195-3p |
| hsa-miR-3692-5p | hsa-miR-622 | hsa-miR-7845-5p | hsa-miR-4459 |
| hsa-miR-3692-3p | hsa-miR-637 | hsa-miR-6796-5p | hsa-miR-4671-5p |
| hsa-miR-3925-5p | hsa-miR-383-3p | hsa-miR-4722-5p |  |
| hsa-miR-2277-3p | hsa-miR-6868-5p | hsa-miR-8072 |  |
| hsa-miR-422a | hsa-miR-6511a-5p | hsa-miR-4475 |  |

**Table S4. Putative miRNAs predicted to binding circCRIM1 by** **StarBase algorithm**

| miRNAs predicted by starBase algorithm | | | |
| --- | --- | --- | --- |
| hsa-miR-145-5p | hsa-miR-320d | hsa-miR-146b-5p | hsa-miR-125b-5p |
| hsa-miR-5195-3p | hsa-miR-942-5p | hsa-miR-4644 | hsa-miR-125a-5p |
| hsa-miR-455-3p | hsa-miR-432-5p | hsa-miR-185-5p | hsa-miR-670-5p |
| hsa-miR-181b-5p | hsa-miR-378c | hsa-miR-4306 | hsa-miR-4319 |
| hsa-miR-181a-5p | hsa-miR-378a-3p | hsa-miR-4436a | hsa-miR-370-5p |
| hsa-miR-181c-5p | hsa-miR-422a | hsa-miR-5000-3p | hsa-miR-376b-3p |
| hsa-miR-181d-5p | hsa-miR-378f | hsa-miR-383-5p | hsa-miR-376a-3p |
| hsa-miR-4262 | hsa-miR-378h | hsa-miR-2115-3p | hsa-miR-455-3p |
| hsa-miR-488-3p | hsa-miR-378i | hsa-miR-665 | hsa-miR-4761-3p |
| hsa-miR-4766-5p | hsa-miR-378d | hsa-miR-2355-5p | hsa-miR-3186-3p |
| hsa-miR-320a | hsa-miR-378b | hsa-miR-499b-5p | hsa-miR-182-5p |
| hsa-miR-320b | hsa-miR-378e | hsa-miR-514a-3p |  |
| hsa-miR-320c | hsa-miR-7153-5p | hsa-miR-514b-3p |  |
| hsa-miR-4429 | hsa-miR-146a-5p | hsa-miR-3140-3p |  |

**Table S5** **Clinical characteristics of NPC patients according to high and low circCRIM1 expression**

| **Characteristics** | **No. of patients** | **Expression of circCRIM1** | | ***P* value** |
| --- | --- | --- | --- | --- |
|  |  | **Low, n (%)** | **High, n (%)** |  |
| **Age** |  |  |  |  |
| ≤ 45 | 103 | 65 (51.2) | 38 (41.8) | 0.169 |
| > 45 | 115 | 62 (48.8) | 53 (58.2) |  |
| **Sex** |  |  |  |  |
| Male | 168 | 94 (74.0) | 74 (81.3) | 0.206 |
| Female | 50 | 33 (26.0) | 17 (18.7) |  |
| **WHO type** |  |  |  |  |
| II | 10 | 5 (3.9) | 5 (3.9) | 0.745 |
| III | 208 | 122 (96.1) | 86 (96.1) |  |
| **VCA-IgA** |  |  |  |  |
| < 1:80 | 19 | 8 (6.3) | 11 (12.1) | 0.135 |
| ≥ 1:80 | 199 | 119 (93.7) | 80 (87.9) |  |
| **EA-IgA** |  |  |  |  |
| < 1:10 | 33 | 19 (15.0) | 14 (15.4) | 0.931 |
| ≥ 1:10 | 185 | 108 (85.0) | 77 (84.6) |  |
| **T Stage** |  |  |  |  |
| T1-T2 | 30 | 14 (11.0) | 16 (17.6) | 0.166 |
| T3-T4 | 188 | 113 (89.0) | 75 (82.4) |  |
| **N Stage** |  |  |  |  |
| N0-N1 | 119 | 77 (60.6) | 42 (46.2) | **0.034** |
| N2-N3 | 99 | 50 (39.4) | 49 (53.8) |  |
| **TNM Stage** |  |  |  |  |
| III | 126 | 75 (59.1) | 51 (56.0) | 0.657 |
| IV | 92 | 52 (40.9) | 40 (44.0) |  |
| **Distant metastasis** |  |  |  |  |
| No | 157 | 102 (80.3) | 55 (60.4) | **0.001** |
| Yes | 61 | 25 (19.7) | 36 (39.6) |  |
| **Death** |  |  |  |  |
| No | 137 | 93 (73.2) | 44 (48.4) | **< 0.001** |
| Yes | 81 | 34 (26.8) | 47 (51.6) |  |

Abbreviations: WHO type II, differentiated non-keratinizing nasopharyngeal carcinoma; WHO type III, undifferentiated non-keratinizing nasopharyngeal carcinoma; VCA-IgA, viral capsid antigen immunoglobulin A; EA-IgA, early antigen immunoglobulin A; *P* value was determined by χ^2^ or Fisher’s exact tests.

**Table S6. Univariate and multivariable Cox regression analysis of circCRIM1 expression level and survival in NPC patients**

| **Variable** | **Univariate analysis** | | |  | **Multivariate analysis** | | |
| --- | --- | --- | --- | --- | --- | --- | --- |
|  | **HR** | **95% *CI*** | ***P* value** |  | **HR** | **95% *CI*** | ***P* value** |
| **Overall survival** |  |  |  |  |  |  |  |
| circCRIM1 expression (high vs. low) | 2.425 | 1.558-3.774 | **< 0.001** |  | 2.134 | 1.364-3.339 | **0.001** |
| T stage (T3-T4 vs. T1-T2) | 0.876 | 0.483-1.590 | 0.664 |  |  |  |  |
| N stage (N2-N3 vs. N0-N1) | 1.927 | 1.239-2.997 | **0.004** |  | 1.943 | 1.243-3.039 | **0.004** |
| Age (≥ 45 vs. < 45 years) | 1.668 | 1.064-2.613 | **0.026** |  | 1.607 | 1.017-2.539 | **0.042** |
| Gender (Female vs. male) | 0.447 | 0.236-0.845 | **0.013** |  | 0.486 | 0.255-0.925 | **0.028** |
| VCA IgA (≥ 1：80 vs. < 1:80) | 1.099 | 0.505-2.387 | 0.812 |  |  |  |  |
| EA IgA (≥ 1：10 vs < 1:10) | 1.096 | 0.593-2.024 | 0.77 |  |  |  |  |
| **Disease-free survival** |  |  |  |  |  |  |  |
| circCRIM1 expression (high vs. low) | 2.112 | 1.380-3.232 | **0.001** |  | 1.915 | 1.248-2.941 | **0.003** |
| T stage (T3-T4 vs. T1-T2) | 0.621 | 0.361-1.070 | 0.086 |  |  |  |  |
| N stage (N2-N3 vs. N0-N1) | 2.304 | 1.494-3.552 | **< 0.001** |  | 2.215 | 1.433-3.423 | **< 0.001** |
| Age (≥ 45 vs. < 45 years) | 1.233 | 0.804-1.890 | 0.336 |  |  |  |  |
| Gender (Female vs. male) | 0.569 | 0.321-1.010 | 0.054 |  |  |  |  |
| VCA IgA (≥ 1：80 vs. < 1:80) | 1.128 | 0.521-2.444 | 0.76 |  |  |  |  |
| EA IgA (≥ 1：10 vs < 1:10) | 1.222 | 0.664-2.250 | 0.519 |  |  |  |  |
| **Distant metastasis-free survival** |  |  |  |  |  |  |  |
| circCRIM1 expression (high vs. low) | 2.413 | 1.447-4.023 | **0.001** |  | 2.107 | 1.258-3.528 | **0.005** |
| T stage (T3-T4 vs. T1-T2) | 0.588 | 0.312-1.106 | 0.1 |  |  |  |  |
| N stage (N2-N3 vs. N0-N1) | 2.408 | 1.434-4.044 | **0.001** |  | 2.272 | 1.348-3.829 | **0.002** |
| Age (≥ 45 vs. < 45 years) | 1.336 | 0.802-2.227 | 0.266 |  |  |  |  |
| Gender (Female vs. male) | 0.328 | 0.141-0.763 | **0.01** |  | 0.344 | 0.148-0.802 | **0.013** |
| VCA IgA (≥ 1：80 vs. < 1:80) | 1.421 | 0.516-3.917 | 0.497 |  |  |  |  |
| EA IgA (≥ 1：10 vs < 1:10) | 1.543 | 0.702-3.393 | 0.28 |  |  |  |  |

Abbreviations: VCA-IgA, viral capsid antigen immunoglobulin A; EA-IgA, early antigen immunoglobulin A; HR, hazard ratio; NS, not significant. Bold values indicate *P* < 0.05, *P* value was determined by Cox regression analysis.

**Table S7. Primers and RNA sequences used in this study**

| **List of oligonucleotide sequences** | **5'→ 3'** |
| --- | --- |
| **Primers for RT-qPCR** | |
| circCRIM1-F/Divergent | CCCGGACAGCTATGAAACTC |
| circCRIM1-R/Divergent | GCAGCCAGCAATAAGGTTTT |
| Convergent-circCRIM1-F | CCGCTGTGAAGTCCAGTTCT |
| Convergent-circCRIM1-R | CTGGGTAAGGGACAGCACTC |
| circCRIM1-F (for FFPE tissues) | GGTTGCTGTACTTTGCCAACA |
| circCRIM1-R (for FFPE tissues) | AAACCAAGCAGTTGGTCATCA |
| CRIM1-F | GGTTCCTGTTGTGCTCTTGT |
| CRIM1-R | TGCCAAGAATCAAGTTGCAGATAA |
| U3 snoRNA-F | CCACGAGGAAGAGAGGTAGC |
| U3 snoRNA-R | CACTCAGACCGCGTTCTCTC |
| GAPDH-F/Convergent | AACGGATTTGGTCGTATTGG |
| GAPDH-R/Convergent | TTGATTTTGGAGGGATCTCG |
| Divergent-GAPDH-F | CCAATACGACCAAATCCGTT |
| Divergent-GAPDH-R | CGAGATCCCTCCAAAATCAA |
| β-actin-F | GCATGGGTCAGAAGGATTCC |
| β-actin-R | AGGATGCCTCTCTTGCTCTG |
| FOXQ1-F | ATCTCCATCAAACGTGCCTT |
| FOXQ1-R | GCAGGCTTCGCAAAGAAACT |
|  |  |
| **Primers for plasmid construction** | |
| LUC-circCRIM1-F | CCGCTCGAGATGAGAACTGGACTGATGACCAACT |
| LUC-circCRIM1-R | AGCTTTGTTTAAACCTTGTTGGCAAAGTACAGCAACCAT |
| LUC-FOXQ1 3'UTR-F | AGCTTTGTTTAAACGACTTTGCACTTTGAATCCA |
| LUC-FOXQ1 3'UTR-R | ATAAGAATGCGGCCGCTGATGTACTTAGGGGGGAGG |
|  |  |
| **siRNAs** |  |
| si-circCRIM1-1 | TTTGCCAACAAGATGAGAA |
| si-circCRIM1-2 | CAACAAGATGAGAACTGGA |
| si-FOXQ1 | CGAGTACCTCATGGGCAAG |

* F, forward; R, reverse.
